# Four and a Half LIM Domains 1b (Fhl1b) Is Essential for Regulating the Liver versus Pancreas Fate Decision and for β-Cell Regeneration

**DOI:** 10.1371/journal.pgen.1005831

**Published:** 2016-02-04

**Authors:** Jin Xu, Jiaxi Cui, Aranzazu Del Campo, Chong Hyun Shin

**Affiliations:** 1 School of Biology and the Parker H. Petit Institute for Bioengineering and Bioscience, Georgia Institute of Technology, Atlanta, Georgia, United States of America; 2 Max Planck Institute for Polymer Research, Mainz, Germany; Cincinnati Children's Hospital Medical Center, UNITED STATES

## Abstract

The liver and pancreas originate from overlapping embryonic regions, and single-cell lineage tracing in zebrafish has shown that Bone morphogenetic protein 2b (Bmp2b) signaling is essential for determining the fate of bipotential hepatopancreatic progenitors towards the liver or pancreas. Despite its pivotal role, the gene regulatory networks functioning downstream of Bmp2b signaling in this process are poorly understood. We have identified *four and a half LIM domains 1b* (*fhl1b*), which is primarily expressed in the prospective liver anlage, as a novel target of Bmp2b signaling. *fhl1b* depletion compromised liver specification and enhanced induction of pancreatic cells from endodermal progenitors. Conversely, overexpression of *fhl1b* favored liver specification and inhibited induction of pancreatic cells. By single-cell lineage tracing, we showed that *fhl1b* depletion led lateral endodermal cells, destined to become liver cells, to become pancreatic cells. Reversely, when *fhl1b* was overexpressed, medially located endodermal cells, fated to differentiate into pancreatic and intestinal cells, contributed to the liver by directly or indirectly modulating the discrete levels of *pdx1* expression in endodermal progenitors. Moreover, loss of *fhl1b* increased the regenerative capacity of β-cells by increasing *pdx1* and *neurod* expression in the hepatopancreatic ductal system. Altogether, these data reveal novel and critical functions of Fhl1b in the hepatic versus pancreatic fate decision and in β-cell regeneration.

## Author Summary

Lineage-specific multipotent progenitors play crucial roles in embryonic development, regeneration in adult tissues, and diseases such as cancer. Bone morphogenetic protein (Bmp) signaling is critical for regulating the cell fate choice of liver versus pancreas, two essential organs of body metabolism. Through transcriptome profiling of endodermal tissues exposed to increased or decreased Bmp2b signaling, we have discovered the zebrafish gene *four and a half LIM domains 1b* (*fhl1b*) as a novel target of Bmp2b signaling. *fhl1b* is primarily expressed in the prospective liver anlage. Loss- and gain-of-function analyses indicate that Fhl1b suppresses specification of the pancreas and induces the liver. By single-cell lineage tracing, we showed that depletion of *fhl1b* caused a liver-to-pancreas fate switch, while *fhl1b* overexpression redirected pancreatic progenitors to become liver cells. At later stages, Fhl1b regulates regeneration of insulin-secreting β-cells by directly or indirectly modulating *pdx1* and *neurod* expression in the hepatopancreatic ductal system. Therefore, our work provides a novel paradigm of how Bmp signaling regulates the hepatic versus pancreatic fate decision and β-cell regeneration through its novel target Fhl1b.

## Introduction

Bone morphogenetic protein (Bmp) signaling plays an essential role in inducing the liver at the expense of *Pdx1*-expressing ventral pancreas and intestinal progenitors in different animal models [1–5]. In murine and zebrafish endodermal progenitors, Bmp signaling activates liver genes by affecting the expression levels of zinc finger transcription factor Gata4 [5,6]. In addition, Bmp signaling induces liver genes epigenetically by activating Smad4, a common-mediator Smad, and recruiting a histone acetyltransferase, p300 [4]. This activation results in histone acetylation at the liver gene regulatory elements [4]. Meanwhile, several studies suggest that Bmp signaling may actively suppress the pancreas gene program. In mice, treating half-embryo cultures with Bmp4 at the 3–4 somite stage inhibited expression of *Pdx1* [3]. Single-cell lineage tracing in zebrafish showed that lateral endodermal cells close to the Bmp2b signal keep *pdx1* expression off, while medial cells distant from the Bmp2b signal turn on *pdx1*, forming a medio-lateral *pdx1* expression gradient [1]. The former differentiates into the liver and the latter gives rise to *pdx1*-positive tissues such as the ventral pancreas and intestine [1]. Consistently, inhibition of BMP signaling was critical for the induction of *PDX1* at the expense of liver gene expression and the consequent generation of INSULIN-secreting β-cells in human embryonic stem cells (hESCs) and zebrafish [7–11]. Activation of Bmp signaling cell-autonomously blocked the induction of β-cells in zebrafish [7]. Nonetheless, the identity of downstream gene regulatory networks of Bmp signaling that specify the liver to the detriment of *Pdx1*-expressing cells remains to be further elucidated. Moreover, the key question of whether Bmp signaling suppresses *Pdx1* expression keeping progenitors competent to differentiate into the liver or directly induces the liver gene program has not yet been answered.

The hepatopancreatic ductal (HPD) system, which consists of the extrahepatic duct (EHD), cystic duct (CD), common bile duct (CBD), and extrapancreatic duct (EPD), connects the liver, gallbladder, and pancreas with the intestine. Amniotes and zebrafish have developmentally and structurally similar HPD systems, both originating from a specific domain within the foregut endoderm that lies between the emerging liver and pancreas [12]. Lineage tracing studies in mammals have revealed that the HPD system and the ventral pancreas, but not the liver, were derived from cells expressing both *Pdx1* and *Sox17*, a master regulator of the pancreaticobiliary ductal system [13]. These data are consistent with the *pdx1* expression in zebrafish [14]. The existence of a progenitor cell population that can differentiate into liver or pancreas cells in the HPD system is supported by the wide spread misdifferentiation of hepatocyte-like and pancreatic-like cells in the HPD system of *fgf10* and *sox9* mutant zebrafish [12,15,16]. Notch signaling and *pdx1* function have been further suggested to play essential roles in the induction of pancreatic endocrine cells from the progenitors in the HPD system and intrapancreatic ducts (IPD) of zebrafish [17]. Intriguingly, the expression of Inhibitor of DNA binding 2 (Id2) protein, a cell-autonomous marker of Bmp signaling activity [18], is excluded in the endocrine pancreas, HPD system, and intrapancreatic ducts [7] which are the tissues that retain the potential to form pancreatic endocrine cells. In a rat pancreatic epithelial cell line, Id2 has been implicated in repressing the function of key pancreatic endocrine transcription factor Neurod, which is essential for endocrine pancreas development [19]. In line with these data, suppression of Bmp signaling by dorsomorphin increased neogenesis of β-cells adjacent to the HPD system in zebrafish. Nonetheless, the underlying mechanisms of how Bmp signaling orchestrates the proper lineage choice of the progenitors in the HPD system await further investigation.

β-cell regeneration can be promoted by either increasing residual β-cell proliferation [20] or stimulating neogenesis of new β-cells from non-β-cells. Non-β-cells include progenitors residing in the extra- and/or intra-pancreatic ductal structures [21], other mature cell types including glucagon-expressing α-cells [22], or digestive enzyme-secreting acinar cells [23]. Although the transcriptional network that regulates β-cell development has been well explored [24,25], the signaling pathways that regulate β-cell regeneration remain largely unknown. Recently, the adenosine signaling pathway has been shown to increase β-cell proliferation during homeostatic control and regeneration of the β-cell mass in a zebrafish model of β-cell regeneration [26]. Nevertheless, compared to several studies that have discovered the origin of newly formed β-cells [27,28], only a few studies have pinpointed extrinsic signaling pathways that can induce *de novo* formation of β-cells during regeneration.

The LIM (LIN-11, ISL-1, and MEC-3) domain is the key protein-protein interaction motif that integrates diverse cellular processes without intrinsic catalytic activity [29,30]. The LIM proteins often contribute to biological activity as molecular adaptors or scaffolds to support the assembly of multimeric protein complexes [31]. The four complete LIM domains with an N-terminal half LIM domain is characteristic of the four-and-a-half LIM (FHL) proteins [32]. These proteins are expressed in a cell- and tissue-specific manner to regulate cellular processes such as proliferation, differentiation, and adhesion/migration. However, little is known about their role in the cell fate choice between the liver and the pancreas and in β-cell regeneration.

Here, by transcriptome analysis, we identified a novel Bmp2b target, *four and a half LIM domains 1b* (*fhl1b*). *fhl1b* is primarily expressed in the prospective liver anlage. Loss- and gain-of-function as well as single-cell lineage tracing analyses indicate that Fhl1b inhibits specification of the pancreas and induces the liver. Moreover, Fhl1b regulates regeneration of insulin-secreting β-cells by directly or indirectly modulating *pdx1* and *neurod* expression in the HPD system.

## Results

### *fhl1b* is a target of the Bmp2b pathway

**Fig 1:**
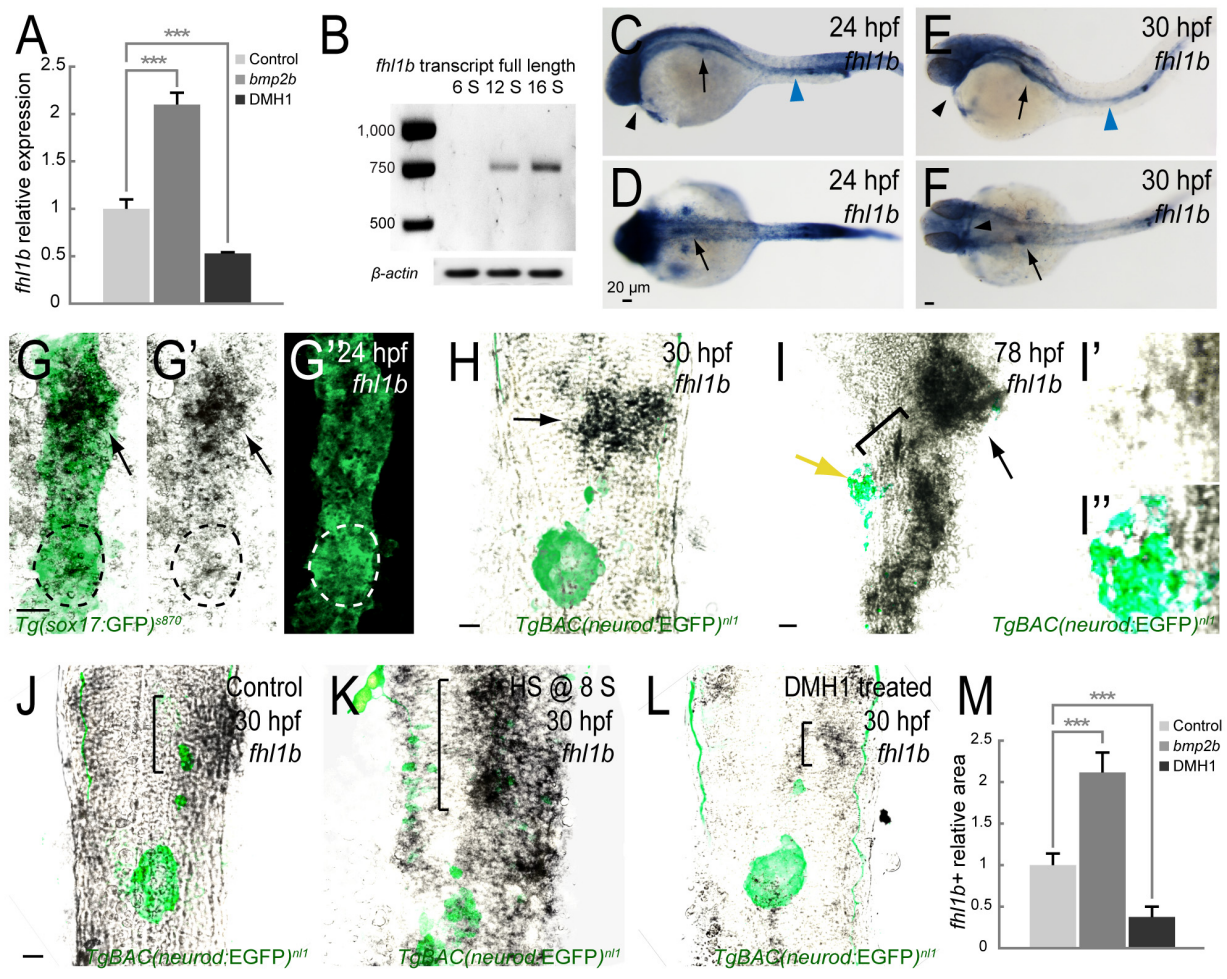
*fhl1b* is a target of the Bmp2b pathway. (A) Quantitative real-time PCR analysis of *fhl1b* in *bmp2b*-overexpressing or DMH1-treated embryos at 20 hours-post-fertilization (hpf). Gene expression was normalized to that of *β-actin* and presented as fold changes (mean±SD) against control expression. Asterisks indicate statistical significance: ***, *P* < 0.001. (B) *fhl1b* full-length transcript, which was detected by RT-qPCR analysis of whole embryos, starts to be expressed from the 12-somite stage onwards. (C-F) Whole-mount *in situ* hybridization showing the expression of *fhl1b* at 24 (C-D) and 30 (E-F) hpf. At 24 hpf (C-D), *fhl1b* is expressed in the anterior part of the endoderm, which corresponds to the prospective liver anlage (black arrows). Additionally, *fhl1b* is expressed in the pronephric duct (blue arrowhead) and heart (black arrowhead). At 30 hpf (E-F), *fhl1b* continues to be highly expressed in the liver (black arrows) and remains to be expressed in the pronephric duct (blue arrowhead) and heart (black arrowheads). (G-G”) Double antibody and *in situ* hybridization staining of *fhl1b* at 24 hpf in *Tg(sox17*:*GFP)*^*s870*^ embryos. *fhl1b* is primarily expressed in the anterior part of the endoderm, which corresponds to the prospective liver anlage (black arrows). White and black dotted circles locate the dorsal pancreatic bud, which gives rise exclusively to the principal islet, a cluster of endocrine cells. A merged view of G’ and G” is shown in G. (H-I”) Double antibody and *in situ* hybridization staining of *fhl1b* in *TgBAC(neurod*:*EGFP)*^*nl1*^ embryos at 30 (H) and 78 (I-I”) hpf. *TgBAC(neurod*:EGFP*)*^*nl1*^ expression marks the posteriorly located pancreatic endocrine cells. At 30 hpf (H), *fhl1b* is highly expressed in the liver (black arrow). At 78 hpf (I-I”), the level of *fhl1b* expression is continuously high in the liver (black arrow) and in patches of cells in the distal intestine, low in the HPD system (black bracket), and absent in most of the pancreatic cells except for a few cells in the periphery of the principal islet (yellow arrow). In the principal islet, *fhl1b* expression is confined to the peripheral boundary and does not significantly overlap with the *TgBAC(neurod*:EGFP*)*^*nl1*^ expression. Magnified images of *fhl1b* and *TgBAC(neurod*:EGFP*)*^*nl1*^expression in the principal islet are shown in I’ and I”. (J-L) Double antibody and *in situ* hybridization staining showing the endogenous expression of *fhl1b* in the liver (black brackets), comparing control (J), *bmp2b*-overexpressing (K), and DMH1-treated (L) *TgBAC(neurod*:*EGFP)*^*nl1*^ embryos at 30 hpf. *fhl1b* expression was greatly expanded when *bmp2b* expression was induced at the 8-somite stage (K), but was reduced in DMH1-treated embryos (L). (M) Quantification of the *fhl1b*-positive *in situ* hybridization signal at 30 hpf. The areas of *fhl1b*-positive signal were selected and measured using Image J with normalization to control. 3 individual embryos were analyzed for each condition. Asterisks indicate statistical significance: ***, *P* < 0.001. G-L, confocal single-plane *in situ* hybridization images combined with the projection images of *Tg(sox17*:GFP*)*^*s870*^ (G-G”) and *TgBAC(neurod*:EGFP*)*^*nl1*^ (H-L) expression, ventral views, anterior to the top. C and E, lateral views, anterior to the left. D and F, dorsal views, anterior to the left. n = 10 per each time point and condition. Scale bars, 20 μm.

In order to uncover novel Bmp2b target genes essential for regulating the fate of bipotential hepatopancreatic progenitors, we performed the transcriptome analysis on endodermal tissues exposed to either increased or decreased levels of Bmp2b signaling (S1A Fig). Total RNA from FACS-sorted *Tg(sox17*:GFP*)*^*s870*^-expressing endodermal cells [33] was used in gene expression profiling analysis. Known genes showing at least a 2-fold (in the case of increased Bmp2b signaling) or a 2.75-fold (in the case of decreased Bmp2b signaling) changes with *p* ≤ 0.05 were clustered by biological processes, which were derived from Gene Ontology analysis using PANTHER (http://www.pantherdb.org/) (S1B Fig). A total of 998 and 1261 genes showed changes in increased (S1 Table) and decreased (S2 Table) Bmp2b signaling, respectively. Among the genes that exhibited at least a 2-fold change in expression in both conditions (S3 Table), *four and a half LIM domains 1b* (*fhl1b*), which encodes a LIM domain only protein (S2B Fig), had a prominent change in expression (S1C Fig). The microarray results were confirmed by reverse transcription quantitative real-time polymerase chain reaction (RT-qPCR) analysis (Fig 1A). The transcription of *fhl1b*, which was detected by RT-qPCR analysis of whole embryos, starts at the 12-somite stage after the endogenous *bmp2b* expression is initiated in the lateral plate mesoderm (LPM) (Fig 1B; [1]). Double antibody and *in situ* hybridization staining in *Tg(sox17*:*GFP)*^*s870*^ embryos revealed that *fhl1b* is primarily expressed in the anterior part of the endoderm, which corresponds to the prospective liver anlage, at 22–24 hours-post-fertilization (hpf) (Fig 1C, 1D, 1G and 1G”, black arrows; early-forming dorsal pancreatic bud, which gives rise exclusively to the principal islet, a cluster of endocrine cells, is marked by white and black dotted circles in G-G”). Additionally, *fhl1b* is expressed in the pronephric duct (Fig 1C and 1E, blue arrowheads) and heart (Fig 1C, 1E and 1F, black arrowheads) from 24 hpf onwards. Double antibody and *in situ* hybridization staining in *TgBAC(neurod*:*EGFP)*^*nl1*^ [34] embryos revealed that *fhl1b* continues to be highly expressed in the liver when the liver has started budding from the medially migrated endodermal rod [35] at 30 hpf (Fig 1E, 1F and 1H, black arrows) and 78 hpf (Fig 1I, black arrow). At 78 hpf, levels of *fhl1b* are also high in patches of cells in the distal intestine (Fig 1I), low in the HPD system (Fig 1I, black bracket), and absent in most of the pancreatic cells except for a few cells in the periphery of the principal islet (Fig 1I, yellow arrow; magnified images of *fhl1b* expression in the principal islet in Fig 1I’ and 1I”). To confirm that Bmp2b signaling regulates *fhl1b*, we examined *fhl1b* expression in the excess or absence of Bmp2b signaling. Compared to control embryos (Fig 1J, black bracket, and Fig 1M), in embryos where *bmp2b* expression was induced at the 8-somite stage, which is before the initiation of endogenous *bmp2b* expression, *fhl1b* expression showed a significant anterior-posterior (A-P) and M-L expansion in the liver (Fig 1K, black bracket, and Fig 1M). Furthermore, suppression of Bmp signaling with DMH1 (a highly selective inhibitor of the BMP type I receptors Alk3 (Bmpr1a) and Alk8 (Acvr1/Alk2)) [36] led to a marked reduction of *fhl1b* expression in the liver at 30 hpf (Fig 1L, black bracket, and Fig 1M). These data indicate that *fhl1b* is a target of Bmp2b signaling and it is primarily expressed in the prospective liver anlage.

Based on the phylogenetic tree of zebrafish Fhl1b and the related proteins in mammals, Fhl1 was selected as the mouse ortholog of zebrafish Fhl1b (S2A Fig). *Fhl1* ablation exacerbated the cardiomyopathy in hypertrophic cardiomyopathy (HCM) mice [37]. Fhl1 shares 61% amino acid identity with Fhl1b (S2B Fig). We examined mRNA and protein expression of Fhl1 in developing mouse embryos. At embryonic day 8.5 (E8.5)- E9.5, *Fhl1* is detected in the foregut endoderm where the liver and the pancreas are derived [3] (S2C Fig). From E10.5 onwards, *Fhl1* is expressed in the liver (S2C Fig). At E14.5, Fhl1 proteins are highly expressed in the Prospero homeobox protein 1 (Prox1)-positive liver cells (S2D–S2D”‘ Fig), whereas their expression is weakly detected in the Prox1-positive pancreatic cells [38,39] (S2E–S2E”‘ Fig). These findings suggest that similar to zebrafish Fhl1b, Fhl1 is expressed in the developing liver. Taken together, these results indicate that the hepatopancreatic expression of Fhl1b and its mouse ortholog Fhl1 is evolutionarily conserved.

### Loss of Fhl1b activity enhances induction of pancreatic cells and compromises liver specification

**Fig 2:**
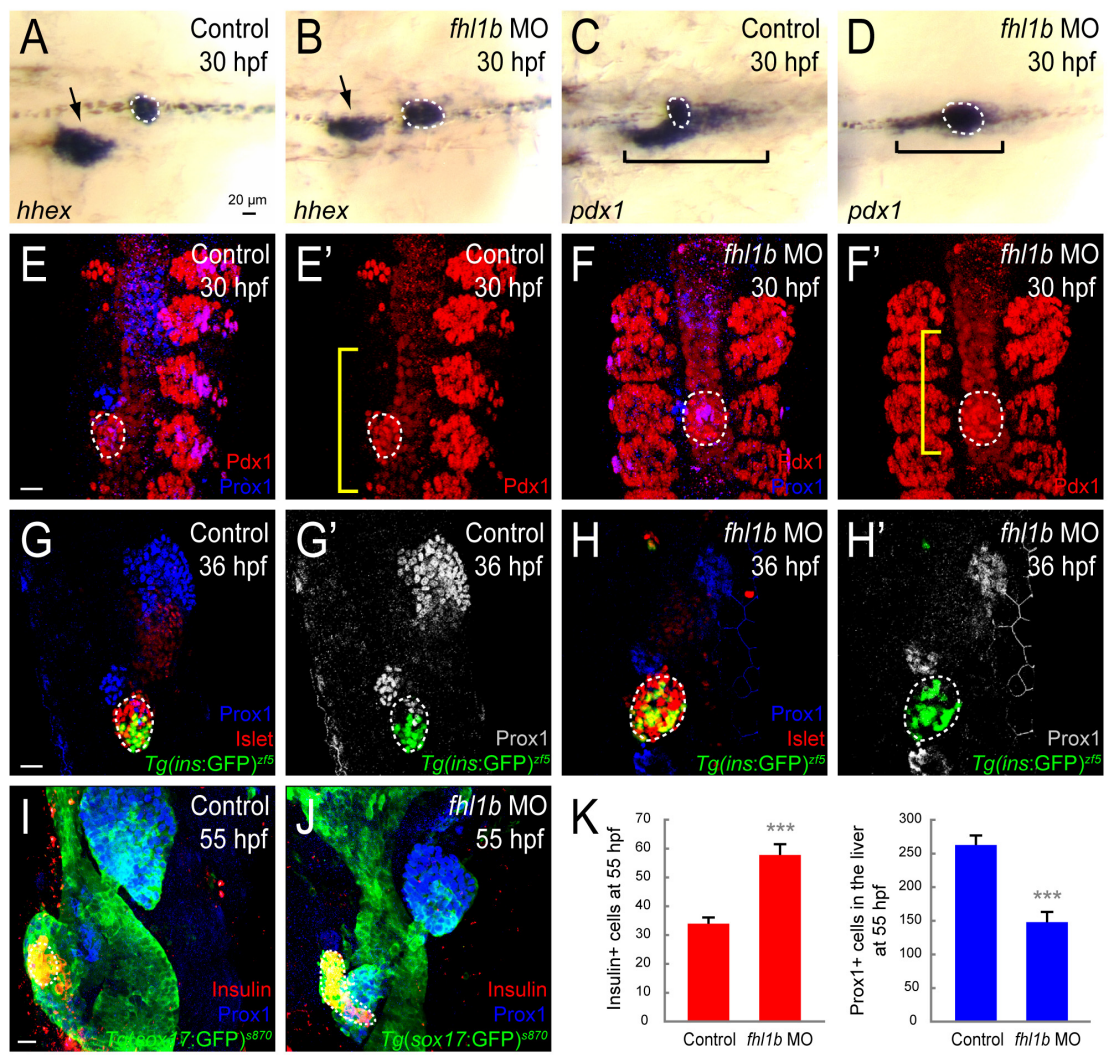
Loss of Fhl1b activity enhances induction of pancreatic cells and compromises liver specification. (A-D) Whole-mount *in situ* hybridization showing the expression of *hhex* (A and B) and *pdx1* (C and D), comparing control embryos (A and C) and *fhl1b* morphants (B and D) at 30 hpf. *hhex* is expressed in the liver (black arrows) and the dorsal pancreatic bud (white dotted circles). *pdx1* is expressed in the developing pancreas including the dorsal pancreatic bud (white dotted circles) and intestine (black brackets), but not in the liver. *hhex* expression was reduced in the liver of *fhl1b* morphants, while expanded in the dorsal pancreatic bud (B). *pdx1* expression in the dorsal pancreatic bud was also expanded, while its expression in the intestinal bulb primordium appeared to be reduced in *fhl1b* morphants (D) compared to that of control embryos (C). (E–F′) Confocal images of control embryos (E and E′) and *fhl1b* morphants (F and F′) at 30 hpf, stained for Pdx1 (red; expression in the dorsal pancreatic bud is outlined by white dotted circles) and Prox1 (blue). The somites are also Pdx1 positive. Compared to that of control embryos (E and E′), in *fhl1b* morphants (F and F′), the Pdx1 expression domain in the dorsal pancreatic bud was expanded, while the Prox1 expression domain was reduced. Note that the Pdx1 expression domain in the intestinal primordium (yellow brackets) appeared to be decreased in morphants (F’). (G-H’) Confocal images of *Tg(ins*:*GFP)*^*zf5*^ control embryos (G and G′) and *fhl1b* morphants (H and H’) at 36 hpf, stained for Islet (red; expression in the dorsal pancreatic bud is outlined by white dotted circles) and Prox1 (blue in G and H; grey in G’ and H’). In *fhl1b* morphants (H and H’), the Prox1 expression domain was greatly reduced, whereas the number of *Tg(ins*:GFP*)*^*zf5*^- and Islet-expressing cells was increased. (I and J) Confocal images of *Tg(sox17*:*GFP)*^*s870*^ control embryos (I) and *fhl1b* morphants (J) at 55 hpf, stained for Insulin (red; outlined by white dotted circles) and Prox1 (blue). *fhl1b* morphants (J) continuously exhibited an enlarged Insulin-expressing β-cell population with a reduced number of Prox1-positive cells in the liver. (K) Quantification of the number (mean±SD) of Insulin-positive cells in the pancreas (red) and Prox1-positive cells in the liver (blue) at 55 hpf. 33.9±2.1 cells were Insulin-positive in control embryos, whereas 57.8±3.6 cells were Insulin-positive in *fhl1b* morphants. 148.0±15.2 cells expressed Prox1 in *fhl1b* morphants, while 262.7±14.0 cells were Prox1-positive in control embryos. Cells in 20 planes of confocal images from 5 individual embryos were counted. Asterisks indicate statistical significance: ***, *P* < 0.001. A-D, dorsal views, anterior to the left. E-J, confocal projection images, ventral views, anterior to the top. Scale bars, 20 μm.

To elucidate the role of Fhl1b in regulating the fate choice of endodermal progenitors, we disrupted the function of *fhl1b* with morpholino oligonucleotides (MOs) either against the splice acceptor site of the second exon, which includes the start codon (MO 1), or against the splice donor site of the third exon (MO 2) (S3A Fig). At 30 hpf, either single MO 1- or MO 2- as well as a mixture of MO 1- and 2-injected embryos (morphants) showed a decrease of the *hhex* [40] expression domain in the liver (Fig 2A and 2B, black arrows), whereas its expression appeared to be expanded in the early-forming dorsal pancreatic bud (Fig 2A and 2B, white dotted circles). The *pdx1* expression domain in morphants was also expanded in the dorsal pancreatic bud (Fig 2C and 2D, white dotted circles), whereas its expression in the intestinal bulb primordium appeared to be reduced (Fig 2C and 2D, black brackets). Immunostaining with the antibodies recognizing Pdx1 and the early liver marker Prospero homeobox protein 1 (Prox1; [35]) in *Tg(sox17*:*GFP)*^*s870*^ embryos [33] (Figs 2E–2F’ and S4A–S4B’) as well as Islet and Prox1 in *Tg(ins*:*GFP)*^*zf5*^ embryos ([41], Fig 2G–2H’), respectively, showed an evident reduction of the Prox1 expression domain in the liver (Fig 2E–2H’), an increase in the number of *Tg(ins*:GFP)^*zf5*^-expressing and Islet-positive pancreatic endocrine cells (Fig 2G–2H’, white dotted circles), and an expansion of the Pdx1-expressing cell population in the dorsal pancreatic bud in morphants at 30–36 hpf (Figs 2E–2F’ and S4A, S4B, S4C and S4D, white dotted circles; 78.3±3.2 cells in controls vs. 101.6±4.1 cells in morphants; n = 5 per condition; *P* = 0.0009). The Pdx1 expression domain in the intestinal primordium appeared to be decreased in morphants (Fig 2E–2F’, yellow brackets). At 55 hpf, morphants continuously exhibited an enlarged Insulin-expressing β-cell population (Figs 2I–2K and S3C, S8I; 33.9±2.1 cells in controls vs. 57.8±3.6 cells in morphants; n = 5 per condition; *P* = 0.00003) with a reduced number of Prox1-positive cells in the liver (Figs 2I–2K and S3C and S8I; 262.7±14.0 cells in controls vs. 148.0±15.2 cells in morphants; n = 5 per condition; *P* = 0.00003). No TUNEL-positive liver cells were observed in *fhl1b* morphants at 48 hpf, suggesting that the small liver observed in morphants was not caused by enhanced cell death (S5A and S5B Fig). In addition to the endodermal phenotypes, morphants displayed pericardial edema and a reduced heart rate from 30 hpf onwards (S3H and S3I Fig, black arrowheads).

To validate the specificities of *fhl1b* MOs, reverse transcription polymerase chain reaction (RT-PCR) was performed. MO 1 and 2 each blocked the endogenous splice sites of *fhl1b* and, as a result, either a deletion of exon 2 (S3B Fig, MO 1, white asterisk) or a formation of a cryptic splice form of exon 3 (S3B Fig, MO 2, white asterisk) occurred. A mixture of both MO 1 and 2 led to the deletion of both exon 2 and 3 (S3B Fig, MO 1& 2, white asterisk). MO-mediated knockdown can often induce apoptosis via aberrant p53 activation. Hence, we performed simultaneous knockdown of *tp53* and *fhl1b* to ameliorate apoptosis induced by MO off-targeting [42]. Single *fhl1b* and double *fhl1b*/*tp53* MO-injected embryos and larvae showed no difference in the phenotypes of small liver (S3E and S3F, white circles, and S3K, S3M and S3N Fig), an increased Insulin-expressing β-cell population (S3E, S3F and S3H–S3J Fig), and pericardial edema (S3H–S3I, black arrowheads) at 55 hpf and 5 days-post-fertilization (dpf). These data indicate that *fhl1b* knockdown phenotypes in the endoderm and heart are independent of the p53 pathway. Throughout this study, *fhl1b* MOs were used as a mixture of MO 1 and 2 (total 4 ng) as each MO caused essentially the same phenotype (S3C Fig), and standard control MO was used as a negative control. Furthermore, co-injection of *fhl1b* mRNA with a mixture of MO 1 and 2 partially rescued the effect of *fhl1b* MO knockdown (S6A–S6D Fig).

To complement *fhl1b* MO knockdown studies, knockout of *fhl1b* was performed by applying the CRISPR/Cas9 nuclease targeting system [43], which has been shown to lead to highly efficient biallelic conversion in somatic cells in zebrafish [44]. We microinjected *cas9* mRNA and two guide RNAs (gRNAs), which were both designed to target overlapping regions in the exon 2 of *fhl1b* (S7A Fig), into one-cell stage embryos. We found that 11.62% of Cas9/gRNA-treated embryos (38 out of 327 embryos) showed an enlarged Insulin-expressing β-cell population (31.6±3.51 cells in controls vs. 45.3±6.0 cells in Cas9/gRNA-treated embryos; n = 5 per condition; *P* = 0.02) with a reduced number of Prox1-positive cells in the liver (265±18.6 cells in controls vs. 164±16.5 cells in Cas9/gRNA-treated embryos; n = 5 per condition; *P* = 0.002) as in *fhl1b* MO knockdown embryos at 55 hpf (S7D–S7F Fig). We randomly selected 4 embryos with these phenotypes and confirmed to contain insertions/deletions (indels) with the T7 endonuclease I (T7EI) assay and Sanger sequencing. T7EI assay revealed that the percent gene modification in the 4 tested embryos was between 21.75% and 31.58% (S7B Fig). Sanger sequencing of these 4 embryos (20–30 PCR amplicons were sequenced for each embryo) confirmed site-specific insertions/deletions (indels) including 2–17 bp deletions or 2–11 bp insertions (S7C Fig). Consistent with the report that Cas9 cuts the target DNA at six base pairs upstream of the protospacer adjacent motif (PAM) [45], all mutations occurred at the 3′ end of the target sequence, further validating the sequence specificity of this targeting process. Taken together, these comparable MO knockdown and CRISPR/Cas9 knockout results suggest that Fhl1b is required for restraining endodermal progenitors from specifying to pancreatic endocrine cells and for the proper induction of the liver.

### Decreased Fhl1b activity augments induction of pancreatic endocrine cells

**Fig 3:**
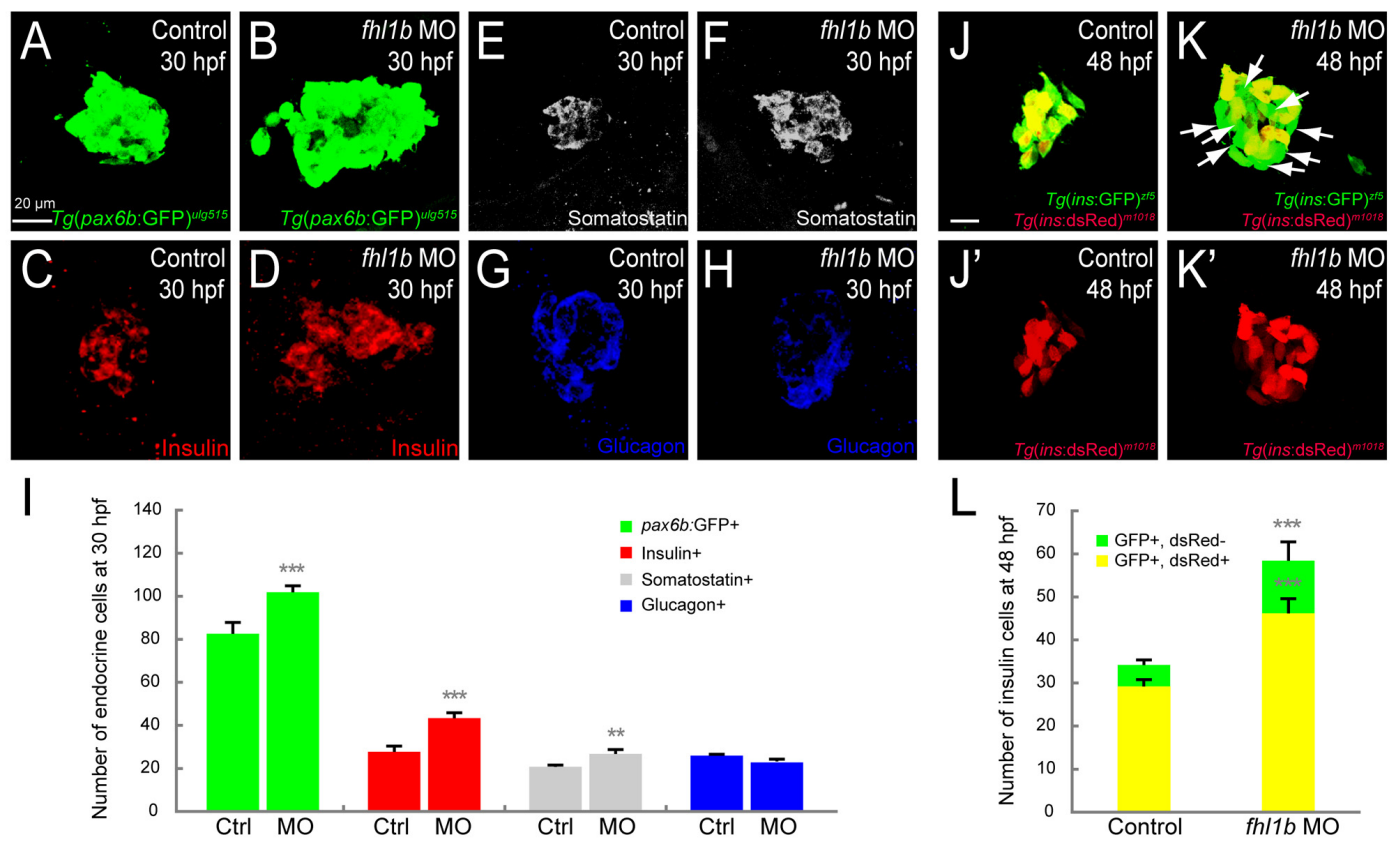
Decreased Fhl1b activity augments induction of pancreatic endocrine cells. (A-H) Confocal images showing *Tg(P0-pax6b*:GFP*)*^*ulg515*^ (A and B, green), Insulin (C and D, red), Somatostatin (E and F, grey), and Glucagon (G and H, blue) expression at 30 hpf, comparing control embryos (A, C, E, and G) and *fhl1b* morphants (B, D, F, and H). The number of *Tg(P0-pax6b*:GFP*)*^*ulg515*^ (B)- and Insulin (D)-expressing cells was significantly increased in *fhl1b* morphants compared to that of control embryos (A and C, respectively). The number of Somatostatin-expressing cells was also increased (F), while that of Glucagon-expressing cells appeared unaffected (H) in *fhl1b* morphants compared to that of control embryos (E and G, respectively). (I) Quantification of the number (mean±SD) of total and individual pancreatic endocrine hormone-expressing cells, comparing that of control embryos and *fhl1b* morphants at 30 hpf. The absolute number of *Tg(P0-pax6b*:GFP*)*^*ulg515*^-, Insulin-, and Somatostatin-expressing cells was increased from 82.6±4.5, 30.6±1.5, and 20.7±0.8, respectively, in control embryos, to 103±2.0, 44.6±2.0, and 26.7±2.0, respectively, in *fhl1b* morphants, while that of Glucagon-expressing cells appeared unaffected (26.5±0.7 cells in controls vs. 24.6±1.5 cells in *fhl1b* morphants). Cells in 20 planes of confocal images from 5 individual embryos were counted. Asterisks indicate statistical significance: **, *P* < 0.01; ***, *P* < 0.001. (J-K’) Confocal images of *Tg(ins*:*GFP)*^*zf5*^*;Tg(ins*:*dsRed)*^*m1018*^ control embryos (J and J’) and *fhl1b* morphants (K and K’) at 48 hpf. Compared with the control embryos (J), *fhl1b* morphants showed an increased number of GFP-only-positive β-cells (K, white arrows). (L) Quantification of the number (mean±SD) of GFP- and dsRed-double positive (yellow) and GFP-only-positive (green) β-cells, comparing that of control embryos and *fhl1b* morphants at 48 hpf. In control embryos, 5.0±0.7 β-cells were GFP-only-positive, while 12.2±2.3 β-cells were GFP-only-positive in *fhl1b* morphants. Cells in 20 planes of confocal images from 5 individual embryos were counted. Asterisks indicate statistical significance: ***, *P* < 0.001. A-H and J-K’, confocal projection images, ventral views, anterior to the top. Scale bars, 20 μm.

To further analyze which pancreatic cell types are induced in *fhl1b* morphants, we first examined the expression of *Tg(P0-pax6b*:GFP*)*^*ulg515*^, a pan-endocrine progenitor reporter [46]. The number of *Tg(P0-pax6b*:GFP*)*^*ulg515*^-expressing cells increased from 82.6±4.5 in controls to 103.2±2.0 in morphants at 30 hpf (Figs 3A, 3B and 3I and S8G; n = 5 per condition; *P* = 0.0009). Next, we investigated which endocrine subpopulation was expanded in the morphants. The number of Insulin-expressing β-cells was increased from 30.6±1.5 in controls to 44.6±2.0 in morphants at 30 hpf (Figs 3C–3D and 3I and S8G; n = 5 per condition; *P* = 0.0004). While the number of Somatostatin-expressing δ-cells was also increased in morphants (Figs 3E, 3F and 3I and S8G; 20.7±0.8 cells in controls vs. 26.7±2.0 cells in morphants; n = 5 per condition; *P* = 0.0033), the number of Glucagon-expressing α-cells appeared unaffected in morphants (Figs 3G, 3H and 3I and S8G; 26.5±0.7 cells in controls vs. 24.6±1.5 cells in morphants; n = 5 per condition; *P* = 0.2). As recently reported [47], Insulin and Glucagon, but not Insulin and Somatostatin, are co-expressed in both control embryos and *fhl1b* morphants at 30 hpf (S8G Fig). The number of these dual-hormone expressing cells was slightly increased in *fhl1b* morphants at 30 hpf (S8G Fig; 8.0±1.0 cells in controls vs. 10.6±1.5 cells in morphants; n = 5 per condition; *P* = 0.03).

A previous report showed that cell-autonomous suppression of Bmp signaling is critical for the induction of endocrine cells derived not only from the early-forming dorsal bud but also from the late-forming ventral bud [7]. In zebrafish, the late-forming ventral pancreas, which mostly generates pancreatic exocrine cells (acinar and duct cells) and endocrine cells, subsequently encapsulates the early-forming, *pdx1*-positive, dorsal pancreas, which gives rise exclusively to the endocrine cells, thus establishing the mature pancreatic structure [14]. To test the role of Fhl1b in the induction of endocrine cells from the ventral bud specifically, we examined the numbers of the newly differentiated ventral bud-derived endocrine cells in *Tg(ins*:*GFP)*^*zf5*^*;Tg(ins*:*dsRed)*^*m1018*^ double transgenic embryos. As dsRed takes 18–22 hours longer than GFP to mature, we can distinguish GFP only (ventral bud-derived) from GFP/dsRed double-positive (dorsal bud-derived) β-cells until at least 60 hpf [48]. At 48 hpf, the number of GFP-only-positive β-cells increased in morphants compared to that of control embryos (Figs 3J–3L and S8H; 5.0±0.7 cells in controls vs. 12.2±2.3 cells in morphants; n = 5 per condition; *P* = 0.0002), suggesting an augmented induction of β-cells from the ventral bud. We further quantified total and subpopulations of pancreatic endocrine cells at 72 hpf. The number of *Tg(P0-pax6b*:GFP*)*^*ulg515*^-, Insulin-, and Somatostatin-expressing cells was increased from 98.6±3.0, 32.3±2.0, and 28.7±1.4, respectively, in control embryos, to 132.0±5.2, 54.8±3.5, and 40.0±2.1, respectively, in morphants (S8A–S8G Fig; n = 5 per condition; *P* = 0.0003, *P* = 0.0003, *P* = 0.006, respectively), while the number of Glucagon-expressing cells appeared unaffected (S8C, S8F and S8G Fig; 28.6±1.1 cells in controls vs. 27.3±1.5 cells in morphants; n = 5 per condition; *P* = 0.2). Altogether, these data suggest that Fhl1b is required for restricting the induction of pancreatic endocrine cells, specifically Insulin- and Somatostatin-expressing cells, from endodermal progenitors.

### Increased Fhl1b activity suppresses specification of pancreatic cells and induces liver

**Fig 4:**
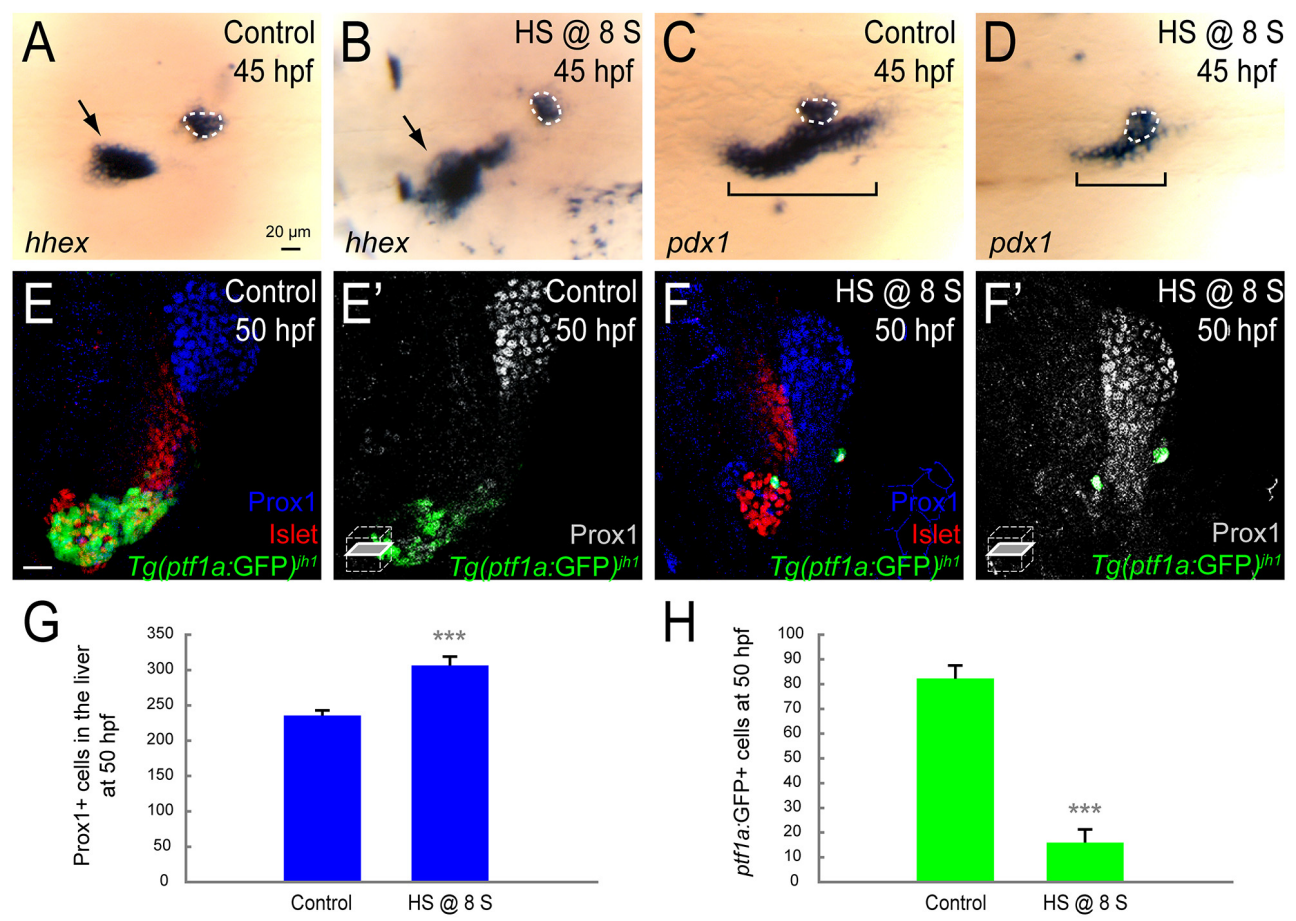
Increased Fhl1b activity suppresses specification of pancreatic cells and induces liver. (A-D) Whole-mount *in situ* hybridization showing the expression of *hhex* (A and B) and *pdx1* (C and D), comparing control embryos (A and C) and *fhl1b-*overexpressing embryos (B and D, heat shock applied at the 8-somite stage) at 45 hpf. *hhex* is expressed in the liver (black arrows) and the dorsal pancreatic bud (white dotted circles). *pdx1* is expressed in the developing pancreas including the dorsal pancreatic bud (white dotted circles) and intestine (black brackets), but not in the liver. When *fhl1b* expression was induced at the 8-somite stage, *hhex* expression was greatly expanded in the liver (B, black arrow), while *pdx1* expression in the developing gut was reduced (D, black bracket). *hhex* and *pdx1* expression in the dorsal pancreatic bud in *fhl1b*-overexpressing embryos was comparable to that of control embryos. (E-F’) Confocal images showing Islet (red), Prox1 (blue in E and F; grey in E’ and F’), and *Tg(ptf1a*:GFP*)*^*jh1*^ (green) expression at 50 hpf, comparing control embryos (E and E’) and *fhl1b*-overexpressing embryos (F and F’, heat shock applied at the 8-somite stage). When *fhl1b* expression was induced at the 8-somite stage (F and F’), the Prox1 expression domain was expanded, whereas *Tg(ptf1a*:GFP*)*^*jh1*^ expression was drastically reduced. (G) Quantification of the number (mean±SD) of Prox1-positive cells in the liver at 50 hpf. 235.5±7.3 cells were Prox1-positive in control embryos, while 306.5±12.6 cells were Prox1-positive in *fhl1b*-overexpressing embryos (heat shock applied at the 8-somite stage). Cells in 20 planes of confocal images from 5 individual embryos were counted. Asterisks indicate statistical significance: ***, *P* < 0.001. (H) Quantification of the number (mean±SD) of *Tg(ptf1a*:GFP*)*^*jh1*^-expressing cells in the exocrine pancreas at 50 hpf. The number of *Tg(ptf1a*:GFP*)*^*jh1*^-expressing cells decreased from 82.2±6.4 in control embryos to 16.0±5.2 in *fhl1b-*overexpressing embryos (heat shock applied at the 8-somite stage). Cells in 20 planes of confocal images from 5 individual embryos were counted. Asterisks indicate statistical significance: ***, *P* < 0.001. A-D, dorsal views, anterior to the left. E and F, confocal projection images; E’ and F’, confocal single-plane images, ventral views, anterior to the top. Scale bars, 20 μm.

In a converse experiment, we assessed the effects of ectopic expression of *fhl1b* on liver and pancreas induction. We overexpressed *fhl1b* using a heat-inducible transgene, *Tg(hsp*:*fhl1b; hsp*:*GFP)*^*gt3*^. In response to heat shock, robust ectopic expression of GFP was observed in a variety of tissues throughout the embryos without any discernible body phenotype. Concurrent expression of *fhl1b* all over the embryos was confirmed with whole-mount *in situ* hybridization. When *fhl1b* expression was induced at the 8-somite stage, the initial time point of *pdx1* expression in the pancreatic exocrine and intestinal progenitors and before the beginning of endogenous *fhl1b* expression, *hhex* expression domain was greatly expanded in the liver at 45 hpf (Fig 4A and 4B, black arrows). In these embryos, *pdx1* expression was significantly reduced in the intestinal bulb primordium and ventral pancreas, which gives rise mainly to the pancreatic exocrine cells, intestine cells, and a few endocrine cells (Fig 4C and 4D, black brackets). *pdx1* expression in the dorsal pancreatic bud appeared unaffected (Fig 4C and 4D, white dotted circles), consistent with the previous data that the lineage of this bud is specified primarily during the gastrulation stage [1]. To determine whether specification of pancreatic exocrine cells is affected in *fhl1b*-overexpressing embryos, we examined the expression of *Tg(ptf1a*:GFP)^*jh1*^ [49], which is largely restricted to the developing exocrine pancreas [50], along with Prox1, which is highly expressed in the liver and developing exocrine pancreas at 50 hpf. Compared to control embryos, we found that in the embryos where *fhl1b* expression was induced at the 8-somite stage, *Tg(ptf1a*:GFP)^*jh1*^expression was almost completely eliminated whereas the Prox1 expression domain was markedly expanded, suggesting that virtually all Prox1-expressing cells are liver cells (Figs 4E–4F’ and S9A). Quantification showed that while 235.5±7.3 cells were Prox1-positive in control embryos, 306.5±12.6 cells expressed Prox1 in *fhl1b*-overexpressing embryos (Figs 4G and S9B; n = 5 per condition; *P* = 0.000067). In contrast, the number of *Tg(ptf1a*:GFP*)*^*jh1*^-expressing cells was decreased from 82.2±6.4 in controls to 16.0±5.2 in *fhl1b-*overexpressing embryos (Figs 4H and S9B; n = 5 per condition; *P* = 0.000004). These results suggest that Fhl1b is sufficient to inhibit specification of pancreatic exocrine cells and induce the liver.

### Fhl1b regulates the patterning and subsequent fate of the medial and lateral endodermal progenitors

**Fig 5:**
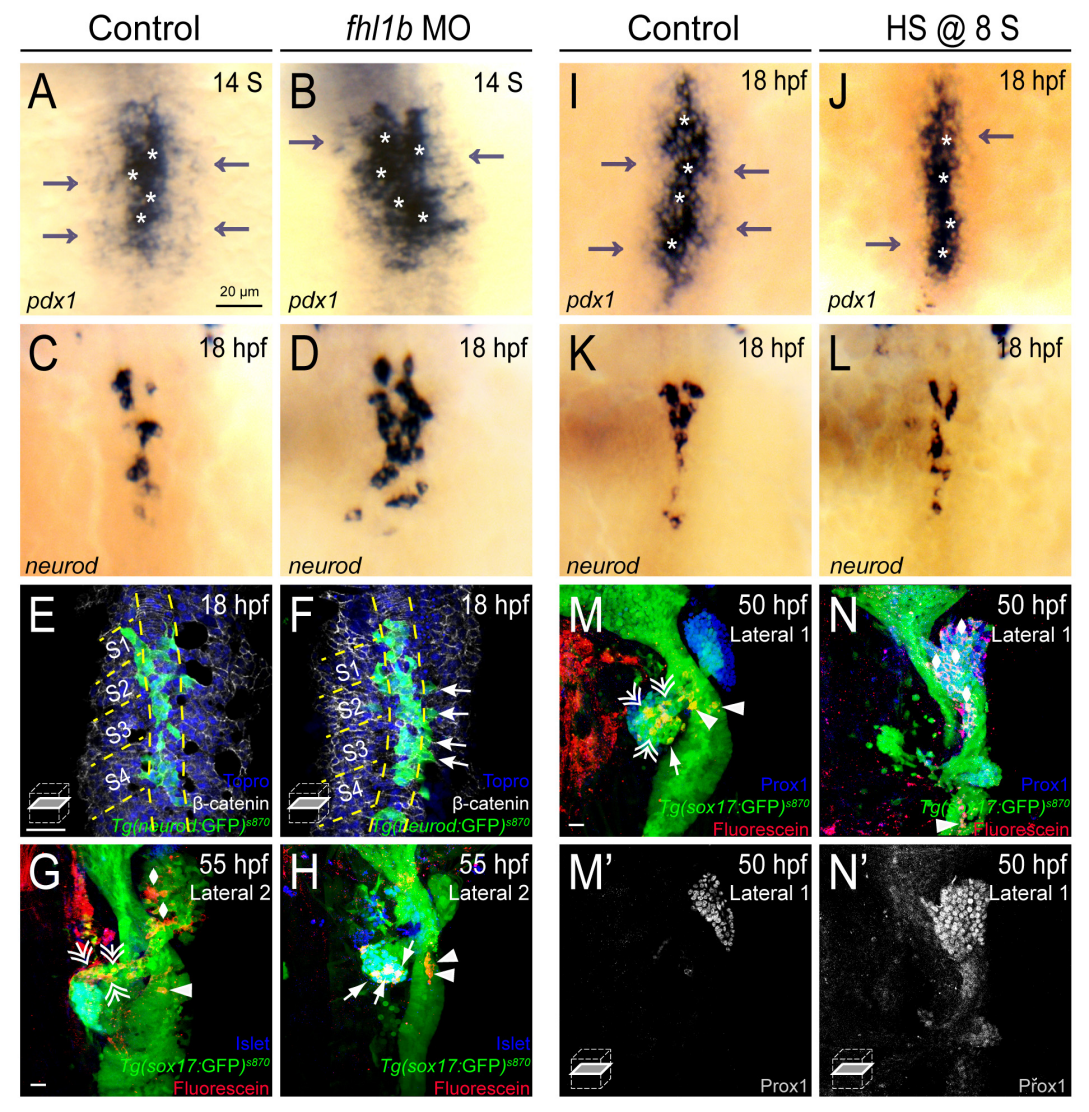
Fhl1b regulates the patterning and subsequent fate of the medial and lateral endodermal progenitors. (A-D) Whole-mount *in situ* hybridization showing the expression of *pdx1* (A and B) and *neurod* (C and D), comparing that of control embryos (A and C) and *fhl1b* morphants (B and D) at the 14-somite stage (A and B) and 18 hpf (C and D). *pdx1* is expressed at high levels in the most medial cells (white asterisks) and at low levels in the lateral cells (gray arrows). *neurod* is expressed in the high-level *pdx1*-expressing cells. In *fhl1b* morphants, high levels of *pdx1* (white asterisks) and *neurod* expression were expanded laterally (B and D). (E-F) Ventral confocal images showing *TgBAC(neurod*:EGFP*)*^*nl1*^, β-catenin (white), and Topro (blue) at 18 hpf (the notochord is outlined by yellow dashed lines). Somites are numbered from anterior to posterior (S1-S4). (E) In control embryos, *TgBAC(neurod*:EGFP*)*^*nl1*^-expressing cells are located close to the notochord. (F) Ectopic *TgBAC(neurod*:EGFP*)*^*nl1*^-expressing cells were found in lateral endodermal regions in *fhl1b* morphants (white arrows). (G and H) Confocal images of *Tg(sox17*:*GFP)*^*s870*^ embryos at 55 hpf, stained for uncaged-Fluorescein (red) and Islet (blue). In control embryos (G), lateral 2 (L2) cells gave rise to the liver (white rhombi), intestine (white arrowhead), and exocrine pancreas (white double arrows), but rarely gave rise to the endocrine pancreas. In *fhl1b* morphants (H), L2 cells contributed to the Islet-positive pancreatic endocrine cells (white arrows), but not to the liver or exocrine pancreas. (I-L) Whole-mount *in situ* hybridization showing the expression of *pdx1* (I and J) and *neurod* (K and L) at 18 hpf, comparing control embryos (I and K) and *fhl1b*-overexpressing embryos (J and L, heat shock applied at the 8-somite stage). In embryos induced to overexpress *fhl1b* at the 8-somite stage (J and L), *neurod* and high levels of *pdx1* expression (white asterisks in J) were maintained, while low levels of *pdx1* expression (gray arrows) were reduced. (M-N’) Confocal images of *Tg(sox17*:*GFP)*^*s870*^ embryos at 50 hpf, stained for uncaged-Fluorescein (red) and Prox1 (blue in M and N; grey in M’ and N’). In control embryos (M and M’), lateral 1 (L1) cells gave rise to the exocrine pancreas (white double arrows) and the intestine (white arrowheads), but not to the liver. In embryos induced to overexpress *fhl1b* at the 8-somite stage (N and N’), L1 cells mostly contributed to the Prox1-positive liver cells (white rhombi). A-D and I-L, dorsal views, anterior to the top. G-H and M-N, confocal projection images; E-F, M’, and N’, confocal single-plane images, ventral views, anterior to the top. Scale bars, 20 μm.

To determine the role of Fhl1b in the M-L patterning of endodermal progenitors, which is essential for the fate decision of liver versus pancreas [1], we first examined the *pdx1* gradient in the endodermal sheet of *fhl1b*-depleted embryos. From the 14-somite stage onwards, morphants showed a dramatic lateral expansion of the *pdx1* expression domain (Fig 5A and 5B). The expression domain of *neurod*, which marks pancreatic endocrine progenitor cells that express high levels of *pdx1* (corresponding to the cells with white asterisks in Fig 5A and 5B; [1,51]), was markedly expanded (Fig 5C and 5D). Furthermore, multiple *TgBAC(neurod*:EGFP*)*^*nl1*^-expressing cells were found even in the lateral part of the endodermal sheet, which normally gives rise to the liver, exocrine pancreas, and intestine (Fig 5E and 5F, white arrows) [1]. Next, we performed single-cell lineage tracing experiments to examine possible cell fate changes caused by modulation of Fhl1b activity. *Tg(sox17*:*GFP)*^*s870*^ embryos were injected at the one-cell stage with the photoactivatable lineage tracer CMNB-caged fluorescein dextran conjugate, and single endodermal cells at 3 different M-L positions (medial, lateral 1, and lateral 2) at the level of somite 2 were uncaged using a 405nm laser at the 6–8 somite stage. In consistent with earlier data [1], in control embryos, lateral 2 cells at the level of somite 2 predominantly gave rise to the exocrine pancreas, intestine, and liver, but rarely to the endocrine pancreas (Figs 5G and S10C–S10E and S10F (as L2) and S10H; in 1 out of 10 control embryos lateral 2 cells gave rise to the endocrine pancreas). In every *fhl1b-*depleted embryo, lateral 2 cells contributed to the pancreatic endocrine cells (Figs 5H and S10D and S10F (as fhl1b MO L2) and S10H; n = 10). Assessment of exocrine pancreas development and differentiation by analyzing the expression of *Tg(ptf1a*:GFP*)*^*jh1*^, which labels developing exocrine pancreatic cells, as well as that of *Tg*(*fabp10a*:DsRed;*ela3l*:EGFP)^*gz15*^ [52], which marks differentiated hepatocytes and pancreatic acinar cells, showed a reduced number of pancreatic exocrine cells at 72 and 96 hpf (S11A–S11D Fig). These data suggest that depletion of Fhl1b function results in the conversion from no/low to high *pdx1*-expressing cells, leading to a significant increase in the number of pancreatic endocrine cells along with a concomitant compromise of the development of liver and pancreatic exocrine cells, which are derivatives of no and low *pdx1*-expressing cells [1].

Conversely, we examined the *pdx1* gradient in *fhl1b*-overexpressing embryos. In *Tg(hsp*:*fhl1b; hsp*:*GFP)*^*gt3*^ embryos in which *fhl1b* expression was induced at the 8-somite stage, medial cells, as their counterpart in control embryos, exhibited high levels of *pdx1* (Fig 5I–5J, white asterisks). Consistently, *neurod* expression appeared unaffected (Fig 5K and 5L). In contrast, lateral cells exhibited greatly reduced levels of *pdx1* compared to that of control embryos (Fig 5I–5J, gray arrows), demonstrating that *fhl1b* overexpression during the post-gastrulation stage led to a decrease of *pdx1* expression in the pancreatic exocrine and intestinal progenitors. Next, a single lateral 1 cell in *Tg(hsp*:*fhl1b; hsp*:*GFP)*^*gt3*^ embryos was heat- shocked and uncaged at the 6–8 somite stage. In every embryo where *fhl1b* expression was induced at the 6–8 somite stage, lateral 1 cells contributed to the liver (Figs 5N and S10D and S10G (as HS @ 8s L1) and S10H; n = 11). However, in most control embryos lateral 1 cells only gave rise to the pancreas and intestine, but not to the liver (Figs 5M and S10B and S10D and S10E and S10G (as L1) and S10H; 1 out of 10 control embryos showed contribution of lateral 1 cells to the liver). These results indicate that augmentation of Fhl1b activity decreases *pdx1* expression levels in pancreatic exocrine and intestinal progenitor cells, leading them to become liver cells.

As previously reported [1], the medial cells at the 6–8 somite stage, which express high levels of *pdx1*, give rise mostly to pancreatic endocrine cells (S10A and S10D and S10E and S10H Fig; n = 11), indicating an early fate restriction of these cells primarily during the gastrulation stage. Intriguingly, forced induction of *fhl1b* during the gastrulation stage led to a significant reduction in the number of high and low *pdx1*-expressing cells resulting in a decrease in the number of Insulin-expressing cells and pancreatic exocrine cells (S12A–S12G Fig). Taken together, these results suggest that Fhl1b plays an essential role in determining the precise patterning of medial and lateral endodermal progenitors by directly or indirectly modulating the levels of *pdx1* expression for proper fate choice of liver versus pancreas.

### Relationship between Bmp2b, Fhl1b, and Id2a in fate choice of liver versus pancreas

Our data indicate that Fhl1b is a novel physiological effector of Bmp2b signaling that regulates the adequate fate choice for liver and pancreas. To investigate the epistatic relationship between Bmp2b signaling and Fhl1b, we induced *bmp2b* expression at the 8-somite stage in the presence or absence of *fhl1b*. As previously reported [1], in *bmp2b*-overexpressing embryos, the Prox1 expression domain in the liver was significantly expanded (S13B Fig), whereas the number of Islet-positive pancreatic endocrine cells appeared unaffected (S13B Fig; red; dorsal pancreatic bud is outlined by white dotted circle). We found that the majority of *bmp2b*-overexpressing *fhl1b* morphants exhibited an enlarged Islet-positive pancreatic endocrine cell population (S13D Fig, 80%; red; dorsal pancreatic bud is outlined by white dotted circle) with a reduced number of Prox1-positive cells in the liver (S13D Fig, 80%) as in *fhl1b* morphants (S13C Fig), whereas a small portion of *bmp2b*-overexpressing *fhl1b* morphants restored the developmental defects of the liver and pancreatic endocrine formation (S13D Fig, 20%). These results suggest that Fhl1b is a critical mediator of Bmp2b signaling in governing the liver versus pancreas fate decision. Furthermore, these data raise the possibility of other effector(s) of Bmp2b signaling that may act in concert with Fhl1b in this process. Hence, we analyzed the function of Id2, which has been shown to suppress the function of Neurod [19] and an immediate target of Bmp signaling [18]. Zebrafish have two *id2* genes: *id2a* and *id2b* [53]. Only *id2a* is expressed in the liver from 30 hpf onwards [53]. We conducted loss-of-function analyses using published *id2a* MO [54]. While *id2a* morphants showed a decrease of the *hhex* expression domain in the liver (S13F Fig, black arrow), its expression in the dorsal pancreas appeared unaffected at 30 hpf (S13F Fig, white dotted circle). Consistently, the *pdx1* expression domain in *id2a* morphants was comparable to that of control embryos (S13J Fig). Double *id2a*/*fhl1b* morphants exhibited synergistically more severe defects in liver formation (S13H Fig, black arrow) than that of single *id2a* (S13F Fig, black arrow) or single *fhl1b* morphants (S13G Fig, black arrow), whereas the dorsal pancreas of double morphants (S13H and S13L Fig, white dotted circles) phenocopied that of single *fhl1b* morphants (S13G and S13K Fig, white dotted circles). The expression of *id2a* in the liver biliary epithelial cells of morphants was comparable to that of control embryos at 72 hpf (S13M and S13N Fig). These data suggest that Id2a is required for hepatic outgrowth, not for the fate decision of liver versus pancreas. Given our results showing incomplete penetrance of phenotype in *fhl1b* morphants (S3C Fig) and restoration of the liver and pancreatic endocrine formation defects in a small portion of *bmp2b*-overexpressing *fhl1b* morphants (S13D Fig, 20%), these data indicate that other effector(s) of Bmp2b signaling may also function to regulate the liver versus pancreas fate decision at least in part (S13O Fig), while Fhl1b plays a major role to govern this process.

### Modulation of Fhl1b activity regulates the capacity of β-cell regeneration

**Fig 6:**
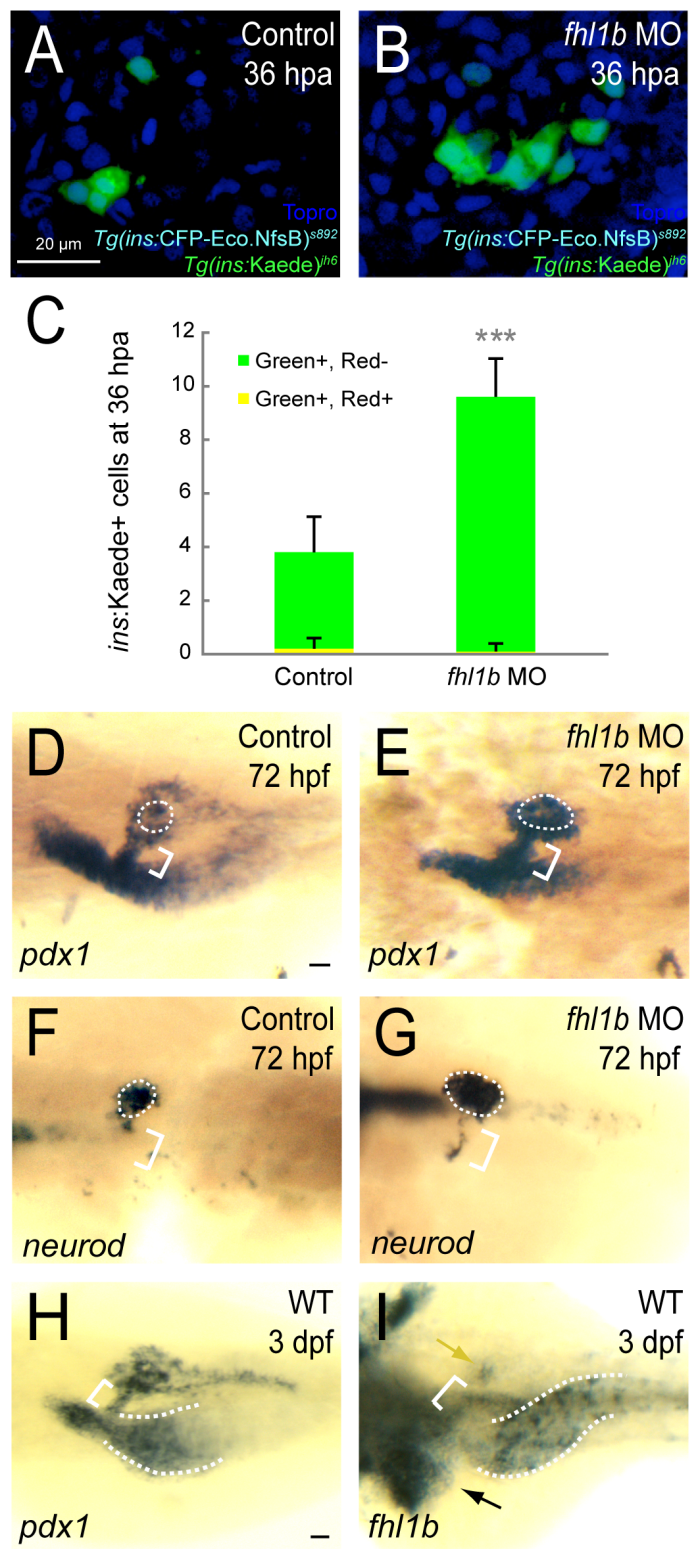
Reduction of Fhl1b activity enhances the capacity of β-cell regeneration. (A and B) Confocal images of [*Tg(ins*:*CFP-NTR)*^*s892*^; *Tg(ins*:*Kaede)*^*jh6*^] control larvae (A) and *fhl1b* MO-injected (B) larvae at 36 hours-post-ablation (hpa) stained with Topro (blue). A greater number of β-cells regenerated in recovering *fhl1b*-MO injected larvae (B) compared to that of control larvae (A). (C) Quantification of the number (mean±SD) of regenerated and survived β-cells. After photoconversion and ablation, the survived β-cells are red and green (yielding a combined color of yellow), whereas the newly formed β-cells are green only. 3.8±1.3 β-cells were green-only-positive in control recovering larvae, while 9.6±1.4 β-cells expressed as green-only in *fhl1b*-MO injected recovering larvae. Almost no β-cells survived the ablation in both the control and *fhl1b*-MO injected recovering larvae. Cells in 20 planes of confocal images from 10 individual larvae were counted. Asterisks indicate statistical significance: ***, *P* < 0.001. (D-G) Whole-mount *in situ* hybridization showing the expression of *pdx1* (D and E) and *neurod* (F and G) at 72 hpf, comparing control embryos (D and F) and *fhl1b* morphants (E and G). *pdx1* is expressed in the pancreas including the principal islet (white dotted circles), the HPD system (white brackets), and the proximal intestine, but not in the liver. *neurod* is expressed mainly in the principal islet (white dotted circles) with slight expression in the HPD system (white brackets). *pdx1* (E) and *neurod* (G) expression in the principal islet and the HPD system was greatly increased in *fhl1b* morphants. (H-I) Whole-mount *in situ* hybridization showing the expression of *pdx1* (H) and *fhl1b* (I) in wild-type embryos at 3 dpf. (H) *pdx1* is expressed in the pancreas including the principal islet, the HPD system (white bracket), and the proximal intestine (white dotted line), but not in the liver. (I) *fhl1b* is expressed at high levels in the liver cells (black arrow), which never express *pdx1*, whereas the HPD system (white bracket) expresses low levels of *fhl1b*. Most pancreatic cells except for a few cells in the principal islet (yellow arrow) do not express *fhl1b*. The distal intestine also expresses *fhl1b* (white dotted lines). A-B, confocal projection images, ventral views, anterior to the top. D-I, dorsal views, anterior to the left. Scale bars, 20 µm.

Given the critical role of Fhl1b in restricting the induction of pancreatic endocrine cells, we investigated whether altering Fhl1b activity changes β-cell regeneration efficiency. Using *Tg*(*ins*:*CFP-Eco*.*NfsB*)^*s892*^ (abbreviated as *Tg*(*ins*:*CFP-NTR*)^*s892*^) [55] together with *Tg(ins*:*Kaede)*^*jh6*^ [56], we compared the β-cell regeneration efficiency in control vs. *fhl1b* MO*-*injected larvae. We first converted the fluorescence of the Kaede protein from green to red by exposing the larvae to UV light. This conversion permanently marked all β-cells that were present before the ablation step. We then treated the larvae from 84−108 hpf with metronidazole (MTZ) to ablate the β-cells. In this set-up, newly formed β-cells express green-fluorescent Kaede only, whereas β-cells that survive the ablation co-express red- and green-fluorescent Kaede. We observed that a greater number of green-only β-cells regenerated in *fhl1b* MO-injected recovering larvae than in control recovering larvae (Figs 6A–6C and S14A; 3.8±1.3 cells per islet in controls vs. 9.6±1.4 cells per islet in *fhl1b* MO-injected larvae; n = 10 per condition; *P* = 0.00000005). Conversely, we overexpressed *fhl1b* using *Tg(hsp*:*fhl1b; hsp*:*GFP)*^*gt3*^ in conjunction with *Tg*(*ins*:*CFP-NTR*)^*s892*^ and *Tg(ins*:*Kaede)*^*jh6*^ to measure the regenerative efficiency of β-cells in control vs. *fhl1b*-overexpressing larvae. We found that the number of regenerated β-cells significantly decreased when *fhl1b* was induced at 50 hpf (S14A Fig; 3.8±1.3 cells per islet in controls vs. 1.5±0.5 cells per islet in *fhl1b*-overexpressing larvae; n = 10 per each condition; *P* = 0.0008). We further examined the underlying mechanism of how Fhl1b modulates the efficiency of β-cell regeneration. At 72 hpf, the number of Islet-positive cells in or adjacent to the HPD system dramatically decreased after inducing *fhl1b* at 50 hpf even in the presence of Fgf receptor inhibitor SU5402, which induces ectopic Islet1-positive cells in the HPD system [7] (S15A–S15C’ Fig). Conversely, at 72 hpf, *fhl1b* morphants showed a dramatic increase of *pdx1* and *neurod* expression in the principal islet (Fig 6D–6G, white dotted circles) and in the HPD system (Fig 6D–6G, white brackets). In line with these expression data, in recovering *fhl1b* MO-injected larvae, multiple regenerating β-cells were found at the junction between the pancreas and the HPD system marked with 2F11 [57] (S14C–S14C” Fig, white arrows). Intriguingly, *fhl1b* and *pdx1* exhibit a reciprocal expression pattern in control embryos at 3 dpf. The level of *fhl1b* expression is high in the liver (Fig 6I, black arrow) and in patches of cells in the distal intestine (Fig 6I, white dotted lines), low in the HPD system (Fig 6I, white bracket), and absent in most pancreatic cells except for a few cells in the principal islet (Fig 6I, yellow arrow). Double antibody and *in situ* hybridization staining in *Tg(ins*:*GFP)*^*zf5*^ embryos at 3 dpf showed that in the principal islet, *fhl1b* expression is confined to the peripheral boundary and does not overlap with the centrally located β-cells (S14D Fig, yellow arrow) nor does with the δ-cells (S14E Fig, black arrowheads) but partially with a small number of α-cells (S14F Fig, black arrowheads). The *pdx1* level of expression is high in the proximal intestine (Fig 6H, white dotted lines) and in most pancreatic cells, moderate in the HPD system (Fig 6H, white bracket), and absent in the liver (Fig 6H). These results indicate that the antagonistic interplay between *fhl1b* and *pdx1* may affect β-cell regeneration by directly or indirectly modulating *pdx1* and *neurod* expression in the HPD system.

Previous studies showed that glucose is crucial for β-cell differentiation and regeneration [47,58] and acts as a potent β-cell mitogen [59–61]. To test the possibility of whether Fhl1b regulates β-cell regeneration by affecting liver-derived glucose production, we measured free glucose levels. At 3 dpf, prior to MTZ treatment, there was no significant difference in free glucose levels between control/WT, *fhl1b*-MO injected, and *fhl1b*-overexpressing larvae (S14G Fig). Free glucose levels were dramatically elevated after β-cell ablation, but were recovered to a great extent from 5–7 dpf in MTZ-treated, MTZ/*fhl1b* MO-injected, and MTZ/*fhl1b*-overexpressing larvae (S14G Fig). Importantly, normal levels of free glucose were recovered significantly faster in the MTZ/*fhl1b* MO-injected larvae (S14G Fig, green line) than in the MTZ-treated (S14G Fig, red line) or MTZ/*fhl1b*-overexpressing larvae (S14G Fig, purple line). Furthermore, MTZ/*fhl1b*-overexpressing larvae still had increased levels of free glucose at 7 dpf (S14G Fig, purple line) compared to MTZ-treated (S14G Fig, red line) or MTZ/*fhl1b* MO-injected larvae (S14G Fig, green line). Taken together, these data suggest that the activity of Fhl1b on the HPD system, rather than the liver-derived glucose production, can modulate the efficiency in restoration of functional β-cells.

## Discussion

**Fig 7:**
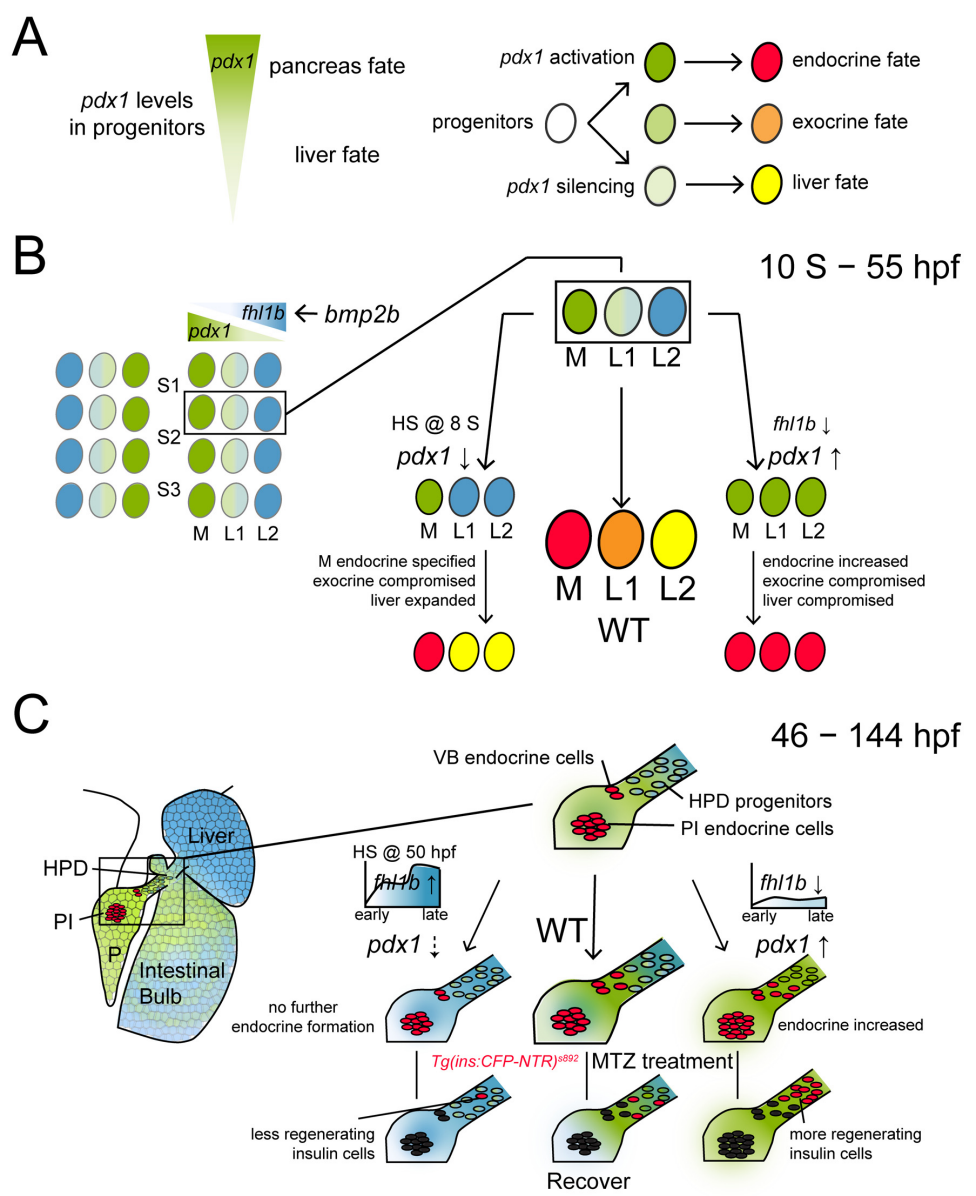
Fhl1b is essential for regulating the cell fate choice of liver versus pancreas and for β-cell regeneration. Schematic model for the role of Fhl1b in lineage specification and in β-cell regeneration. The expression of *fhl1b* and *pdx1* is color-coded as blue and green, respectively. (A) Endodermal progenitors experience different levels of *pdx1* regulating their fates as pancreatic endocrine (high levels of *pdx1*), pancreatic exocrine (low levels of *pdx1*), or liver (*pdx1* silencing) cells. (B) From the 12-somite stage onwards, single endodermal cells in the lateral 2 position (L2) between somites 1 and 3 in the endodermal sheet give rise not only to pancreatic exocrine cells and intestinal cells, but also to liver cells, functioning as bipotential hepatopancreatic progenitors. *bmp2b*, which is expressed in the lateral plate mesoderm, induces *fhl1b* expression in the prospective liver anlage. This may form a reciprocal gradient of *fhl1b*-*pdx1*. Decreased Fhl1b function leads to an increase in levels of *pdx1* expression in lateral 1 and 2 cells, causing a hepatic and pancreatic exocrine to a pancreatic endocrine fate switch. Conversely, augmentation of Fhl1b activity at the initial time point of *pdx1* expression in the pancreatic exocrine and intestinal progenitors (HS @ 8 S) causes a decrease in levels of *pdx1* expression in pancreatic exocrine and intestinal progenitor cells, leading them to become liver cells. The lineage of the most medial cells, which express high levels of *pdx1* and subsequently give rise to pancreatic endocrine cells, is specified primarily during the gastrulation stage. (C) At later embryonic/larval stages, the HPD system comprises a progenitor cell population that can differentiate into pancreatic endocrine cells and liver cells. At these stages, *fhl1b* (color-coded as blue) shows a reciprocal expression pattern with *pdx1* (color-coded as green). Liver cells, which never express *pdx1*, express high levels of *fhl1b*, while the HPD system expresses low levels of *fhl1b*. The distal intestine also expresses *fhl1b*, whereas most pancreatic cells except for a few cells in the principal islet do not express *fhl1b*. In normal development, the ventral bud-derived β-cell formation initiates between 40–46 hpf (VB endocrine cells). Overexpression of *fhl1b* inhibited further induction of pancreatic endocrine cells and β-cell regeneration by potentially inhibiting *pdx1* expression in the HPD system, whereas suppression of *fhl1b* increased *pdx1* and *neurod* expression in the HPD system, augmenting pancreatic endocrine cell formation and subsequent β-cell regeneration. Abbreviations: S1, somite 1; S2, somite 2; S3, somite 3; M, medial; L1, lateral 1; L2, lateral 2; HPD, hepatopancreatic duct; PI, principal islet; P, pancreas; VB, ventral bud; WT, wild-type; MTZ, metronidazole.

In this study, we analyzed the essential functions of a novel Bmp2b downstream effector Fhl1b in the hepatic versus pancreatic fate decision and in β-cell regeneration. In bipotential hepatopancreatic progenitors from the 12-somite stage onwards, Fhl1b regulates the proper cell fate choice of the liver over the pancreas by directly or indirectly modulating the discrete levels of *pdx1* expression (Fig 7A and 7B). *fhl1b* depletion compromised liver and exocrine pancreas specification and enhanced induction of pancreatic endocrine cells, causing a hepatic-to-pancreatic endocrine fate switch. Conversely, *fhl1b* overexpression at the 8-somite stage promoted liver specification and inhibited induction of pancreatic cells, redirecting pancreatic progenitors to become liver cells. In the progenitors residing in the HPD system at later stages, Fhl1b regulates induction of pancreatic endocrine cells and regeneration of β-cells (Fig 7C). Suppression of *fhl1b* increased *pdx1* and *neurod* expression in HPD progenitors, augmenting pancreatic endocrine cell formation and β-cell regeneration, whereas overexpression of *fhl1b* inhibited induction of pancreatic endocrine cells and β-cell regeneration.

Previously, we showed that there is a medial-lateral *pdx1* “gradient” in the endodermal sheet in zebrafish [1]. The most medial cells with high levels of *pdx1* mainly gave rise to pancreatic endocrine cells, whereas lateral 1 cells with low levels of *pdx1* gave rise to pancreatic exocrine cells and intestinal cells, as well as a few pancreatic endocrine cells. Some lateral 2 cells without *pdx1* expression populate the liver. Consistently in mice, a hypomorphic allele with targeted deletion of a *cis*-regulatory region of *Pdx1* in combination with a protein-null allele has demonstrated that the level of *Pdx1* gene activity is differentially required for the proper development of the pancreas and subsequent lineage allocation of the pancreatic endocrine cells [62]. While homozygous mutants of the *Pdx1* enhancer region deletion resulted in severe impairment of endocrine maturation, but normal formation of acinar tissue, heterozygous mice showed an islet size similar to that of wild type mice with abnormally more α and pancreatic polypeptide- producing PP cells, but fewer differentiated β-cells. These findings support the possibility that conversion of common endocrine precursors to β-cells relies on a high-level of *Pdx1* expression. Our studies show that depletion of *fhl1b* resulted in the conversion from no/low to high *pdx1*-expressing cells, which is marked by *neurod* expression. This conversion led to a significant increase in the number of pancreatic endocrine cells, especially β-cells, and compromised the development of liver and pancreatic exocrine cells which are derivatives of no and low *pdx1*-expressing cells. In these embryos, lateral 2 cells contributed frequently to pancreatic endocrine cells. Conversely, *fhl1b* overexpression at the post-gastrulation stage (i.e. 8-somite stage) caused a decrease in the number of low *pdx1*-expressing cells, leading to the induction of the liver at the expense of the exocrine pancreas. In these embryos, lateral 1 cells contributed primarily to liver cells. When *fhl1b* was overexpressed during the gastrulation stage, it led to a decrease in the number of low and high *pdx1*-expressing cells, resulting in a subsequent reduction in the number of pancreatic exocrine cells and Insulin-expressing β-cells. These data confirm the critical role of Fhl1b in directly or indirectly modulating *pdx1* levels to coordinate the medio-lateral patterning of the endodermal sheet for proper induction of the liver and pancreas. Intriguingly, the numbers of β- and δ-cells were increased, whereas the number of α-cells appeared unaffected in *fhl1b* morphants. These results are consistent with previous data that Bmp receptor *alk8* MO-injected donor cells mainly gave rise to β- and δ-cells, but rarely to α-cells [7]. It has been shown that β/δ-cell versus α-cell fate is differentially regulated by Pax4 and Arx [63]. Moreover, overexpression of *Pdx1* eliminated glucagon mRNA and protein and promoted the expression of β-cell specific genes, while induction of dominant-negative *Pdx1* resulted in differentiation of β-cells into α-cells in the rat insulinoma cell line [64]. Hence it is plausible to speculate that Fhl1b is an essential mediator of Bmp signaling by directly or indirectly regulating the discrete levels of *pdx1* expression for precise lineage allocation of the pancreatic endocrine progenitors. Interestingly, we found that in a portion of embryos from 30 hpf onwards, *fhl1b* is also expressed in the *TgBAC(neurod*:EGFP*)*^*nl1*^-expressing cells. Therefore, it is possible to hypothesize that after serving as an essential effector for the hepatic versus pancreatic fate decision, Fhl1b may function further to fine-tune the lineage allocation of the specified pancreatic endocrine cells. As LIM proteins often function as molecular adaptors or scaffolds to support the assembly of multimeric protein complexes [31], it will be intriguing to determine whether Fhl1b directly modulates *pdx1* expression by facilitating the formation of a novel protein complex that is involved in either mediator-or chromatin-mediated gene expression control.

Previous studies have suggested the plasticity of cells in the HPD system, where differentiation into a specific lineage is suppressed by Fgf10 and Sox9b in zebrafish [12,15,16]. Furthermore, expression analysis of Id2 has shown that Bmp signaling is blocked and/or excluded in HPD and non-HPD tissues (principal islets and intra-pancreatic ducts) that retain the potential to form pancreatic endocrine cells [7]. Our data provide the intriguing evidence that Bmp2b signaling controls the induction of pancreatic endocrine cells from the HPD system by inhibiting *pdx1* expression through its effector Fhl1b. The reciprocal expression pattern of *fhl1b* and *pdx1* further supports the suppressive effect of Fhl1b on *pdx1* expression. At 3 dpf, liver cells, which never express *pdx1* in lineage tracing analyses in mice [62,65] and in zebrafish [1], express high levels of *fhl1b*, while the HPD system expresses low levels of *fhl1b*. Consistently, the proximal intestine, which has been shown to have marked plasticity [12], expresses low levels of *fhl1b*. Most pancreatic cells do not express *fhl1b* except for a few cells in the principal islet. Intriguingly, these few pancreatic cells are located in the peripheral boundary of the principal islet and partially overlap with a small number of α-cells, not with the core β-cells, which maintain a high-level of *pdx1* expression. Manipulating this antagonistic interplay may direct a common endodermal progenitor pool towards pancreatic endocrine, specifically β-cell, fate by directly or indirectly modulating distinct levels of *pdx1* expression.

While the intrinsic transcriptional network that regulates β-cell development is well identified [24,25], the extrinsic signaling pathways that control β-cell regeneration remain largely elusive. For the first time, our studies suggest that Bmp signaling plays an essential role in the regeneration of β-cells, in part by directly or indirectly modulating *pdx1* and *neurod* expression in the HPD system through its regulator Fhl1b. Our loss-of-function analyses of Fhl1b during development imply that increased formation of endocrine progenitors may primarily lead to enhanced β-cell regeneration. In line with this hypothesis, in β-cell ablated *fhl1b* MO-injected larvae, multiple regenerated β-cells were found at the junction between the pancreas and the HPD system, specifically at the extrapancreatic duct (EPD). However, because of the low expression levels of *fhl1b* in a small population of α-cells, we were not able to exclude the compelling possibility that *fhl1b* depletion lead to the occurrence of high *pdx1*^+^ α-cells, augmenting β-cell regeneration. In mouse and zebrafish models of β-cell regeneration, Pdx1 is detected in α-cells during α-to β-cell transdifferentiation [22,47,66], contrary to its normal detection in β-cells [67]. In contrast to Bmp signaling, adenosine signaling, one of the few signals that has been shown to function during β-cell depletion in zebrafish [26], plays a significant role in regulating β-cell mass during regeneration, but not under normal conditions. Careful dissection of extrinsic signals and intrinsic factors acting on a specific aspect of β-cell regeneration will allow us to perform individual or combinatorial therapies to pinpoint the most valid regeneration strategy.

Our findings of Bmp2b regulation of Fhl1b suggest a new paradigm of how Bmp signaling regulates the cell fate choice of liver versus pancreas and β-cell mass. Despite the long-standing focus on the active role of Bmp signaling on the liver gene program through both genetic and epigenetic regulation [4–6], the link between Fhl1b and *pdx1* expression shown in this study suggests that Bmp may function actively to suppress the pancreas gene program to properly modulate liver induction, lineage allocation, and β-cell regeneration. Hence, our data elucidates why effective BMP suppression is critical for the induction of *PDX1* and the subsequent generation of β-cells in human pluripotent stem cells (hESCs) [8–11] and zebrafish endodermal progenitors [7]. A comprehensive understanding of how lineage-specific multipotent progenitors make a developmental choice will shed light on the programming and reprogramming of stem/progenitor cells into specific cell lineages, enabling us to generate functionally relevant cells for clinical utility.

## Materials and Methods

### Ethics statement

This study was approved by the Institutional Animal Care and Use Committee at Georgia Institute of Technology (A13075). All animal work was performed according to procedures approved by the Institutional Animal Care and Use Committee at Georgia Institute of Technology.

### Zebrafish strains

This study was approved by the Institutional Animal Care and Use Committee at Georgia Institute of Technology (A13075). All animal work was performed according to procedures approved by the Institutional Animal Care and Use Committee at Georgia Institute of Technology. Adult fish and embryos were raised and maintained under standard laboratory conditions [68]. We used the following published zebrafish transgenic lines: *Tg(P0-pax6b*:GFP*)*^*ulg515*^ [46], *Tg(ins*:*GFP)*^*zf5*^ [41], *Tg(ins*:*dsRed)*^*m1018*^ (from W. Driever, Freiburg), *TgBAC(neurod*:*EGFP)*^*nl1*^ [34], *Tg(sox17*:*GFP)*^*s870*^ [33], *Tg(hsp70l*:*bmp2b)*^*f13*^ [69], *Tg(fabp10*:*dsRed*, *ela3l*:*GFP)*^*gz12*^ [52], *Tg(ptf1a*:*GFP*)^*jh1*^ [49], *Tg(ins*:*Kaede)*^*jh6*^ [56], *Tg*(*ins*:*CFP-NTR*)^*s892*^ [55], and *Tg(fabp10a*:*CFP-Eco*.*NfsB)*^*gt1*^ [70]. To generate the *Tg(hsp*:*fhl1b; hsp*:*GFP)*^*gt3*^, *fhl1b* coding sequence was amplified (forward: 5’-CCGGAATTCATGGCAAGCCGGTCCAACTG-3’, reverse: 5’-CCGGAATTCTTACAGTTTCTTGGAGCAGTCG-3’) and cloned into a vector containing a multimerized minimal heat shock promoter, which drives *gfp* and *fhl1b* transcription bi-directionally in response to a heat shock [71]. Tol2-mediated transgenesis was achieved as described [72].

### Microarray and phylogenetic analysis

*Tg(sox17*:*GFP)*^*s870*^ embryos were either crossed with *Tg(hsp70l*:*bmp2b)*^*f13*^ to induce overexpression of *bmp2b* at the 8-somite stage or treated with 0.3 μM DMH1. For each condition, 100 embryos were used. At 20 hpf, *sox17*:GFP-positive endodermal cells from dissected zebrafish trunks containing the organ-forming area were isolated by FACS and subjected to transcriptome profiling using the Zebrafish 44K gene expression microarray (Agilent Technologies). Data with an average fold change of 2 (*bmp2b* overexpressing) or 2.75 (DMH1-treated) at *p* ≤ 0.05 were considered for GO analysis using PANTHER (http://www.pantherdb.org/). The phylogenetic tree of zebrafish Fhl1b (NM_199217) was constructed using Phylogeny.fr [73] with mammalian homologous proteins sorted by performing alignment on UniProtKB/Swiss-Prot database.

### Reverse transcription quantitative real-time polymerase chain reaction

Total RNA was extracted using the Trizol Reagent (Invitrogen). cDNA synthesis was performed using Superscript III First-strand Synthesis System (Invitrogen). PCR was conducted using iTaq Universal SYBR Green Supermix in triplicate (Bio-Rad). Optimized primers targeting each gene were designed using Primer3 [74]. The StepONE Plus PCR System (Applied Biosystems) was used to obtain the *C*t value. The relative gene expression of each sample was determined using the comparative *C*t method with *β-actin* as an internal control [75]. The following primers were used: *fhl1b*: forward 5’-GTGAGGAAAGACGAGAAACAAG-3’, reverse 5’-GGCACATCGGAAACAATCAG-3’; *β-actin*: forward 5’-CGAGCTGTCTTCCCATCCA-3’, reverse 5’-TCACCAACGTAGCTGTCTTTCTG-3’; mouse *Fhl1*: forward 5’- ATAAGGTGGGCACCATGTCGG-3’, reverse 5’- GTGATTCCTCCAGATGTGATGG-3’.

### Embryo microinjection

Knockdown of *fhl1b* was performed via injection of individual *fhl1b* MO 1 (2 ng; 5’-CCCGCGAAAAGCTGTGAGAAATAAT-3’) or MO 2 (2 ng; 5’-ATAAATATCTGTCCCCTCACCTGGC-3’) or a combination of MO 1 and 2 (4 ng; Gene Tools, LLC). A standard control MO (4 ng; 5’-CCTCTTACCTCAGTTACAATTTATA-3’) targeting a human beta-globin intron mutation was used as a negative control (Gene Tools, LLC). *id2a* MO (5’ -GCCTTCATGTTGACAGCAGGATTTC-3’) [54] and *tp53* MO (5’- GACCTCCTCTCCACTAAACTACGAT-3’) [42]were purchased from Gene Tools, LLC. 4 ng of *id2a* MO or 2 ng of *tp53* MO was used. The primers annealing to the first (5’- GCAAAACACTTTGCTGTGGC-3’) and the sixth (5’- GCCAGGTTGAGGGAGCATTT-3’) coding exons were used to confirm the specificity of *fhl1b* MOs. Sense-strand-capped *fhl1b-P2A-mCherry* mRNA was synthesized with mMESSAGE mMACHINE kit (Ambion). For rescue experiments, embryos were injected with 200 pg of *fhl1b-P2A-mCherry* mRNA with a mixture of *fhl1b* MO 1 and MO 2.

### *In situ* hybridization and immunohistochemistry

Whole-mount *in situ* hybridization was performed as previously described [76], using the following probes: *pdx1* [77], *neurod* [78], *hhex* [79], and *fhl1b* (template for antisense RNA probe was amplified from embryonic cDNA with the following primers: forward: 5’-CCGCTCGAGATGGCAAGCCGGTCCAACTG-3’, reverse: 5’-ACGGCTGGTCCTGGTAATTC-3’). Immunohistochemistry on whole-mount zebrafish embryos was performed as previously described [12] using the following antibodies: mouse anti-Glucagon (1:100; Sigma), mouse anti-2F11 (1:200; Abcam), mouse anti-β-catenin (1:100; BD Transduction Laboratories), chicken anti-GFP (1:1000; Aves Labs), rabbit anti-Somatostatin (1:100; MP Biomedicals), mouse anti-Islet1/2 (1:10; Developmental Studies Hybridoma Bank (DSHB), clone 39.4D5), guinea pig anti-Insulin (1:100; Sigma), rabbit anti-Prox1 (1:100; Millipore), guinea pig anti-Pdx1 (1:200; gift from C. Wright), rabbit anti-pan-Cadherin (1:1000; Sigma), goat anti-Fluorescein (1:100; Molecular Probes), rabbit anti-Carboxypeptidase (1:100; Rockland), and fluorescently conjugated Alexa antibodies (1:200; Molecular Probes). Nuclei were visualized with TOPRO (1:10000; Molecular Probes). For the TUNEL assay, embryos were fixed in 3% formaldehyde, preincubated in PBST, and then labeled with the TUNEL kit (Roche) for 1 hour at 37°C. For coimmunostaining with Prox1, sections were first incubated with primary antibodies, then with TUNEL solutions, and finally with secondary antibodies. For the detection of mouse Fhl1 protein, the entire gut, including the liver and pancreas of E14.5 mice, was isolated and fixed in 4% paraformaldehyde, then embedded in Tissue-Tek OCT compound (Sakara Finetek). 8 μm cryostat sections were obtained by using a cryostat microtome (Leica CM1520). Immunohistochemistry was performed using the following antibodies: rabbit anti-FHL1 (1:200; Abcam), goat anti-Prox1 (1:20; R&D Systems), and fluorescently conjugated Alexa antibodies (1:200; Molecular Probes). Embryos and sections were mounted in Vectashield (Vector Laboratories) and imaged on a Zeiss LSM 510 VIS confocal microscope.

### *fhl1b* gene disruption with the CRISPR/Cas9 system

The guide RNA (gRNA) targeting sites, which are downstream of the start codon (gRNA 1: 5’- CTGTCGTGAGGACCTCAG-3’, gRNA 2: 5’- AGTGGAAAGAAGTTCGTG-3’), were selected using the online application available at crispr.mit.edu. Complementary oligonucleotides corresponding to the target sequences were annealed as previously described [44]. Annealed oligonucleotides (1 μl) were mixed with 500 ng of the gRNA cloning vector pDR274, 0.5 μl of BsaI-HF, 0.5 μl of T4 DNA ligase, 1 μl of 10× NEB buffer 2, 1 μl of 10× T4 ligase buffer, and water for a total of 10 μl. Digestion and ligation were performed in a single step as previously described [44]. The gRNAs were transcribed using HindIII-digested expression vectors as templates and the MEGAshortscript T7 kit (Life Technologies). The *cas9* mRNA was transcribed using NotI-digested Cas9 expression vector and the mMESSAGE mMACHINE kit (Ambion). The mixture of 1 nl of *cas9* mRNA (300–450 ng/μl) and an individual or a combination of gRNA 1 and 2 (final concentration 12.5 ng/μl) were injected into one-cell stage embryos.

### T7 Endonuclease I (T7EI) assay

The genomic region flanking the target sites was amplified using PCR (forward: 5’-ACTTACACATGAGGGGCTGTG-3’, reverse: 5’- ATAGTCCTTAATGGAAAACATGCTG-3’). A total of 200 ng of the purified PCR products was denatured and re-annealed, as previously described [44] to facilitate heteroduplex formation. The re-annealed products were digested with 10 units of T7 endonuclease I (New England Biolabs) at 37°C for 30 min. The reaction was stopped by adding 1 μl of 0.5 M EDTA. Samples were analyzed by 2% agarose gel. Band intensity was quantified using ImageJ software (National Institutes of Health). Gene modification levels were estimated based on the following equation [80]:

$$\%\;\text{gene modification}=100\times \left(1-{\left(1-\text{fraction cleaved}\right)}^{\frac{1}{2}}\right)\%.$$

### DNA sequencing

For sequencing the target region in injected embryos, the PCR products were cloned into pGEM T-easy vector (Promega). Plasmid DNA was isolated from individual transformants and sequenced.

### Chemical treatment and heat-shock experiment

Embryos were treated with 0.3 μM DMH1 (EMD Chemicals) from 12 hpf to 20 hpf or 3 μM SU 5402 (Tocris Bioscience) from 50 hpf to 72 hpf in egg water. To ablate β-cells, *Tg(ins*:*CFP-NTR)*^*s892*^ embryos were treated with freshly prepared 5 mM metronidazole (MTZ) (Sigma) from 84 hpf to 108 hpf in the dark, followed by 24–48 hours recovery. Before ablation, *Tg(ins*:Kaede*)*^*jh6*^-expressing β-cells were converted from green to red by exposing them to UV light. Control embryos from the same batch were treated with DMSO in egg water. *Tg(hsp*:*fhl1b; hsp*:*GFP)*^*gt3*^ and *Tg(hsp70l*:*bmp2b)*^*f13*^ embryos were heat shocked at various stages by transferring them into egg water pre-warmed at 40°C and 37°C, respectively. After a 30-minute heat shock, embryos were returned into a 28°C incubator and harvested at various stages.

### Synthesis and photochemical properties of caged fluorescein dextran

Caged fluorescein dextran was synthesized by coupling a dextran-spermine conjugate to 5-carboxymethoxy-2-nitrobenzyl (CMNB)-caged carboxyfluorescein succinimidyl ester. Dextran-spermine conjugate was produced from dextran (MW 10 kDa, Molecular Probes). In the final product, 20% glucose units were bonded with spermine (estimated by ^1^H NMR spectroscopy). A mixture of dextran-spermine conjugate (8 mg) and CMNB-caged carboxyfluorescein succinimidyl ester (1 mg, Molecular Probes) in 1 mL of borate buffer (0.1 M, pH 8.5) was added into a tinted tube and reacted on a vortexer at room temperature for 24 hours, protected from light. After the reaction, the solution was poured into a dialysis membrane (14000 cutoff cellulose membrane) and dialyzed with deionized water at 4°C for 1 day. The dialysate was gravimetrically filtered to remove insoluble parts and lyophilized to dryness. A total of 4.6 mg were obtained (yield 57% w/w). The average loading of CMNB-caged carboxyfluorescein on dextran was 3.2 dye molecules per dextran chain (estimated from UV-Visible spectra). Light exposure at 360 nm removed the CMNB cage and rendered the free fluorescein modified dextran. Uncaging was followed in solution by the increase in absorbance in the region of 350–550 nm and the increase in fluorescence emission between 460–700 nm. Apparent quantum yield of uncaging is calculated to be 0.0051 from fluorescence spectra.

### Lineage tracing

*Tg(sox17*:*GFP)*^*s870*^ embryos were injected with 2 nl of 0.5% caged fluorescein dextran and allowed to develop until the 6-somite stage (corresponding to 12 hours-post-fertilization (hpf)). After manual dechorionation, embryos were mounted ventrally in a mold filled with egg water. Using a Nikon Eclipse Ti confocal microscope, we visualized the endodermal sheet in live embryos at the 6–8 somite stage, and the A-P position of endodermal cells was determined by counting somites. Caged-fluorescein was activated in a single endodermal cell in each embryo with a 405 nm laser focused through a 40X objective lens. The uncaged embryos were fixed at various time points and stained with antibodies against GFP, uncaged-Fluorescein, and Islet1 or Prox1.

### Glucose measurements

Glucose measurements were performed 3 times on 10 zebrafish larvae per condition using a fluorescence-based enzymatic detection kit (Biovision Inc.) [26]. The larvae were collected in 1.5 ml microcentrifuge tubes. Excess medium was removed and embryos were frozen on crushed dry ice. After thawing, 200 μl PBS was added and the larvae were homogenized using a hand-held mechanical homogenizer. Reactions were assembled on ice in black, flat bottom 96-well plates (Costar). Standard curves were generated using glucose standard solution (according to instructions) and were included in each assay. To measure glucose in embryo extracts, 15 μl of sample were used. Control reactions without sample lysate were included in each row. Reactions were incubated for 30 minutes at 37°C in the dark. Fluorescence (excitation 535 nm; emission, 590 nm) was measured using a Safire II plate reader equipped with XFLUOR4 software (v 4.51). Fluorescence values were corrected by subtracting measurements from control reactions without sample. Glucose levels were interpolated from standard curves. Each sample was measured in triplicate and each experiment repeated three times.

### Statistical analysis

The *p*-values were calculated using an unpaired two-tailed Student *t*-test with Excel (Microsoft, Redmond, WA).

## Supporting Information

S1 Fig Strategy for the identification of Bmp2b downstream genes associated with the liver versus pancreas fate decision. (A) Bmp2b signaling was pharmacologically or genetically manipulated in *Tg(sox17*:*GFP)*^*s870*^ embryos either by treating with DMH1 or inducing *bmp2b* expression at the 8-somite stage. *Tg(sox17*:GFP*)*^*s870*^ -positive endodermal cells from dissected zebrafish trunks containing the organ-forming area (red rectangle) were isolated by FACS and subjected to transcriptome profiling at 20 hpf. (B) Functional clustering and distribution of known genes identified in the expression profiling with a *p*-value ≤ 0.05 and minimum a 2-fold change in *bmp2b* overexpressing or a 2.75-fold change in DMH1-treated embryos. Fifty-six known genes showed significant changes in both *bmp2b* overexpressing and DMH1-treated embryos. (C) List of genes showing prominent changes in both DMH1-treated and *bmp2b*-overexrpessing conditions.
(TIF)

S2 Fig *Fhl1*, the ortholog of zebrafish *fhl1b*, is expressed in the liver during mouse embryonic development. (A) Phylogenetic tree of zebrafish Fhl1b (highlighted in blue) and the related proteins in mammals. This tree was constructed using Phylogeny.fr with sorted candidates from UniProtKB/Swiss-Prot database. Zebrafish (dr), Mouse (mm), Rat (rn), and Human (hs). (B) Alignment of zebrafish Fhl1b and mouse Fhl1 amino acid sequences. Identical residues are indicated with asterisks. (C-E”‘) Expression of Fhl1 in developing mouse embryos. (C) *Fhl1* full-length transcript is expressed in the mouse foregut endoderm at embryonic day 8.5 (E8.5)-E9.5. From E10.5, *Fhl1* is expressed in the liver. (D-E”‘) Immunofluorescent labeling of Fhl1 in the liver (D-D”‘) and pancreas (E-E”‘) of E14.5 mice (n = 3). (D-D”‘) Fhl1 proteins are highly co-expressed in the Prox1-positive liver cells. (E-E”‘) Fhl1b proteins are weakly detected in the Prox1-positive pancreas cells. To better visualize hepatic and pancreatic Fhl1 expression, magnified images for Prox1 (red; top panel), Fhl1 (green; middle panel), and a merged view (bottom panel) are shown in insets in D’-D”‘ and E’-E”‘, respectively.
(TIF)

S3 Fig Specificity of *fhl1b* morpholinos. (A) Schematic diagram of *fhl1b* genomic structure and targeting positions of *fhl1b* MOs (red lines). Black arrows indicate the position of primers (F and R) used for RT-PCR analysis shown in (B). E1-E6: exon 1 to exon 6. Dark grey, coding regions; Light grey, untranslated regions. (B) RT-PCR analysis of *fhl1b* knockdown efficiency. Both MO 1 and MO 2 blocked the endogenous splice site of *fhl1b* and, as a result, either a deletion of exon 2 (MO 1, white asterisk) or a formation of a cryptic splice form of exon 3 (MO 2, white asterisk) occurred, while a combination of MO 1 and 2 led to deletion of both exon 2 and 3 (MO 1 & 2, white asterisk). (C) The percentages of embryos are given for each single MO or combination of MOs based upon the expression domain of *Tg(ins*:GFP*)*^*zf5*^ in the pancreas and Prox1 in the liver at 55 hpf. The embryos were scored as having a “reduced” or “increased” expression domain when the expression area of each marker was distinctly (> 25%) smaller or larger than that of the control embryos based upon the calculation using ImageJ. (D-F) Fluorescent images of *Tg(ins*:dsRed*)*^*m1018*^ and *Tg(sox17*:GFP*)*^*s870*^ expression showing that the developmental defects of the liver (white dotted circles) and β-cell formation in single *fhl1b* morphants (E) was comparable to double *fhl1b*/*tp53* morphants (F) at 55 hpf (n = 52, control; n = 64, single *fhl1b* morphants; n = 72, double *fhl1b*/*tp53* morphants). (G-I) Bright-field images combined with fluorescent images showing the overall morphology of embryos and *Tg(ins*:dsRed*)*^*m1018*^ expression (red) in control (G), single *fhl1b* morphants (H), and double *fhl1b*/*tp53* morphants (I) at 5 dpf. The enlarged *Tg(ins*:dsRed*)*^*m1018*^ -expressing cell population (white squares and insets) in single *fhl1b* morphants (H) was similar to that in embryos co-injected with *fhl1b* and *tp53* MOs (I). Note that potential off-target ventricle lumen inflation defects in the brain of single *fhl1b* morphants were attenuated by co-knockdown of *tp53* (black arrows), whereas pericardial edema persisted both in single *fhl1b* morphants and double *fhl1b*/*tp53* morphants (black arrowheads). (J) Quantification of the results in G-I. The embryos were scored as having an “increased” expression domain when the expression area of *Tg(ins*:dsRed*)*^*m1018*^ was distinctly (> 25%) larger than that of the control embryos based upon the calculation using ImageJ. (K) Quantification of the number (mean±SD) of Prox1-positive cells in the liver at 55 hpf. 252.6±11.5 cells were Prox1-positive in control embryos, while 151.3±16.2 and 142.3±17.4 cells expressed Prox1 in single *fhl1b* morphants and double *fhl1b*/*tp53* morphants, respectively (*P* = 0.0009 and *P* = 0007, respectively). Cells in 20 planes of confocal images from 5 individual embryos were counted. Asterisks indicate statistical significance: ***, *P* < 0.001. (L-N) Confocal images of control embryos (L), single *fhl1b* morphants (M), and double *fhl1b*/*tp53* morphants (N) at 55 hpf, stained for Prox1 (blue). The reduced Prox1-expressing cell population in single *fhl1b* morphants (M) was similar to that in embryos co-injected with *fhl1b* and *tp53* MOs (N). D-F, dorsal views, anterior to the left. G-I, lateral views, anterior to the right. L-N, confocal projection images, ventral views, anterior to the top. Scale bars: D-F and L-N, 20 μM; G-I, 100 μM.
(TIF)

S4 Fig Loss of Fhl1b activity compromises liver specification and enhances induction of Pdx1-positive cells in the dorsal pancreatic bud. (A-B’) Confocal images of *Tg(sox17*:*GFP)*^*s870*^ control embryos (A and A′) and *fhl1b* morphants (B and B′) at 30 hpf, stained for Pdx1 (red; dorsal pancreatic bud is outlined by white dotted circles) and Prox1 (blue in A and B; grey in A’ and B’). The somites are also Pdx1 positive. Compared to control embryos (A and A′), in *fhl1b* morphants (B and B′), the Pdx1 expression domain in the dorsal pancreatic bud was expanded, while the Prox1 expression domain was significantly reduced. (C-D) Quantification of the number (mean±SD) of Pdx1-positive cells in the pancreas at 30 hpf. Cells in 20 planes of confocal images from 5 individual embryos were counted. Asterisks indicate statistical significance: ***, *P* < 0.001. A-B’, confocal projection images, ventral views, anterior to the top. Scale bar, 20 μm.
(TIF)

S5 Fig Cell death does not contribute to the reduction of liver size upon *fhl1b* depletion. (A-B) TUNEL labeling (red) combined with anti-Prox1 immunostaining (grey) revealed that no TUNEL-positive liver cells were observed both in *fhl1b* morphants and control embryos at 48 hpf. (C-C’) As a control, *Tg(fabp10a*:*CFP-NTR)*^*gt1*^ embryos were used. Treating metronidazole (MTZ) caused apoptosis in a large number of hepatocytes. A-C’, confocal projection images, ventral views, anterior to the top. Scale bar, 20 μm.
(TIF)

S6 Fig *fhl1b* mRNA injection partially rescues the effect of *fhl1b* MO knockdown. (A-C”) The developmental defects of the liver (A-C and white dotted circles in A”-C”) and β-cell formation (A-C and A’-C’) in *fhl1b* morphants (B-B”) could be partially rescued by injection of *fhl1b-P2A-mcherry* mRNA (C-C”), restoring liver size and the β-cell population to a degree comparable to that of control embryos (A-A”) at 55 hpf. Fhl1b translation was monitored by mCherry expression as shown in C. (D) Quantification of the results in A-C”. The embryos were scored as having a “reduced” or “increased” expression domain when the expression area of *Tg(ins*:dsRed*)*^*m1018*^ and *Tg(sox17*:GFP*)*^*s870*^ was distinctly (> 25%) smaller or larger than that of the control embryos based upon the calculation using ImageJ. A-C, bright-field images combined with fluorescent image of *Tg(ins*:dsRed*)*^*m1018*^ and *Tg(sox17*:GFP*)*^*s870*^ expression. A’-C”, fluorescent images of *Tg(ins*:dsRed*)*^*m1018*^ and *Tg(sox17*:GFP*)*^*s870*^ expression. Dorsal views, anterior to the left. Scale bar, 20 μm.
(TIF)

S7 Fig Cas9/gRNAs induces indels in the *fhl1b* locus in zebrafish. (A) Illustration showing the position of two gRNA-targeting sites (red lines) in the *fhl1b* locus in zebrafish. Black arrows indicate the position of primers (F and R) used for sequencing to identify indels shown in (C). (B) Representative T7EI assay showing the efficiency of Cas9-mediated cleavage in a single embryo at 55 hpf. (C) Representative Sanger sequencing results of the PCR amplicons of 4 individual embryos at 55 hpf, showing indels induced by Cas9/gRNA in the targeted *fhl1b* locus. Twenty to thirty clones were sequenced for each embryo. The wild-type sequence is shown at the top with the target sites highlighted in yellow and the PAM sequences (TGG and AGG) highlighted in blue. Deletions are shown as red dashed lines and insertions are highlighted in red. The net change in length caused by each indel is to the right of each sequence (+, insertion; -, deletion). (D-E’) Confocal images of *Tg(ins*:*GFP)*^*zf5*^ control embryos (D and D’) and Cas9/gRNA-induced mutant embryos (E and E’) at 55 hpf, stained for Prox1 (blue in D and E; grey in D’ and E’) and Islet (red; expression in the dorsal pancreatic bud is outlined by white dotted circles). Cas9/gRNA-induced mutant embryos exhibited an enlarged Insulin-expressing β-cell population with a reduced number of Prox1-positive cells in the liver, phenocopying that of the *fhl1b* MO knockdown embryos. (F) Quantification of the number (mean±SD) of Insulin-positive cells in the pancreas (green) and Prox1-positive cells in the liver (blue) at 55 hpf. 31.6±3.5 cells were Insulin-positive in control embryos, whereas 45.3±6.0 cells expressed Insulin in Cas9/gRNA-induced mutant embryos. 164±16.5 cells expressed Prox1 in Cas9/gRNA-induced mutant embryos, while 265±18.6 cells were Prox1-positive in control embryos. Cells in 20 planes of confocal images from 5 individual embryos were counted. Asterisks indicate statistical significance: *, *P* < 0.05, **, *P* < 0.01. D-E’, confocal projection images, ventral views, anterior to the top. Scale bar, 20 μm.
(TIF)

S8 Fig Decreased Fhl1b activity increases the number of pancreatic endocrine cells. (A-F) Confocal images showing *Tg(ins*:dsRed*)*^*m1018*^ (A and D) or Insulin (B-C, E-F, red) expression with *Tg(P0-pax6b*:GFP*)*^*ulg515*^ (A and D, green), Somatostatin (B and E, grey), or Glucagon (C and F, blue) expression at 72 hpf, comparing control embryos (A-C) and *fhl1b* morphants (D-F). The number of *Tg(ins*:dsRed*)*^*m1018*^- or Insulin-expressing cells was significantly increased in *fhl1b* morphants (D-F) compared to that of control embryos (A-C). The number of *Tg(P0-pax6b*:GFP*)*^*ulg515*^- and Somatostatin-expressing cells was also increased (D and E, respectively), whereas that of Glucagon-expressing cells appeared unaffected (F) in *fhl1b* morphants compared to control embryos (A, B, and C, respectively). (G) Quantification of the number (mean±SD) of total and individual pancreatic endocrine hormone-expressing cells, comparing control embryos and *fhl1b* morphants at 30 and 72 hpf. (H) Quantification of the number (mean±SD) of dsRed- and GFP-positive β-cells, comparing control embryos and *fhl1b* morphants at 48 hpf. (I) Quantification of the number (mean±SD) of Insulin-positive cells in the pancreas and Prox1-positive cells in the liver at 55 hpf. A-F, confocal projection images, ventral views, anterior to the top. G-I, cells in 20 planes of confocal images from 5 individual embryos were counted. Scale bar, 20 μm.
(TIF)

S9 Fig Increased Fhl1b activity during the post-gastrulation stage inhibits specification of exocrine pancreas and induces liver. (A) The expression of *Tg(ptf1a*:GFP*)*^*jh1*^ in the exocrine pancreas and Prox1 in the liver (heat shock applied at the 8-somite stage) was examined at 50 hpf and the percentages of embryos were quantified. The embryos were scored as having a “reduced” or “increased” expression when the expression of each marker was distinctly (> 25%) smaller or larger than that of the control embryos based upon the calculation using ImageJ. (B) Quantification of the number (mean±SD) of *Tg(ptf1a*:GFP*)*^*jh1*^-positive pancreatic exocrine cells and Prox1-positive cells in the liver, comparing control embryos and *fhl1b*-overexpressing embryos (heat shock applied at the 8-somite stage) at 50 hpf. Cells in 20 planes of confocal images from 5 individual embryos were counted.
(TIF)

S10 Fig Endodermal progenitors contribute to distinct endodermal tissues based on their M-L position and the activity of Fhl1b. (A-C) Confocal images of *Tg(sox17*:*GFP)*^*s870*^ embryos at 55 hpf, stained for Islet (blue) and uncaged-Fluorescein (red), showing the progeny of the medial (A), lateral 1 (B) and lateral 2 (C) cells. Medial cells (A) mostly gave rise to pancreatic endocrine cells (white arrows). Lateral 1 cells (B) gave rise to pancreatic exocrine (white double arrows), endocrine (white arrow), and intestinal (white arrowheads) cells. Lateral 2 cells (C) gave rise to liver (white rhombi), intestine (white arrowhead), and pancreatic exocrine cells (white double arrow). (D-H) The numbers and the percentages of embryos that showed incorporation into a given tissue type in each specific position, comparing control embryos (D, E, F (as L2), G (as L1), and H) as well as *fhl1b* morphants and embryos induced to overexpress *fhl1b* at the 8-somite stage (D, F (as fhl1b MO L2), G (as HS @ 8s L1), and H). In every *fhl1b-*depleted embryo, lateral 2 cells contributed to the pancreatic endocrine cells (D, F (as fhl1b MO L2), and H), while in control embryos, most of the lateral 2 cells gave rise to the exocrine pancreas, intestine, and liver, but seldom gave rise to the endocrine pancreas (D, E, F (as L2), and H). In control embryos, lateral 1 cells mostly gave rise to pancreatic and intestinal cells, but not to liver cells (D, E, G (as L1), and H), whereas in every *fhl1b*-overexpressing embryo, lateral 1 cells contributed to the liver (D, G (as HS @ 8s L1), and H). Data in each 3-D column (%) in E-G were obtained by summing the number of embryos that showed incorporation into a given tissue type and normalizing it to the total number of embryos examined in each specific position: M, L1 and L2. Colored rectangles in H highlight the most dominant pattern in *fhl1b* morphants (red) and *fhl1b*-overexpressing (blue) embryos. A-C, confocal projection images, ventral views, anterior to the top. Scale bar, 20 μm.
(TIF)

S11 Fig Decreased Fhl1b activity inhibits liver and exocrine pancreas differentiation. (A and B) Confocal images of *Tg(ptf1a*:*GFP*)^*jh1*^ control embryos (A) and *fhl1b* morphants (B), stained for Prox1 (blue) and Islet (red). The expression domain of the Prox1 and *Tg(ptf1a*:GFP)^*jh1*^ was reduced in *fhl1b* morphants (B) compared to that of control embryos (A). (C and D) Confocal images of *Tg(fabp10a*:*RFP*, *ela3l*:*EGFP)*^*gz12*^ control embryos (C) and *fhl1b* MO-injected larvae (D) at 96 hpf. The expression domain of the *Tg(fabp10a*:RFP, *ela3l*:EGFP*)*^*gz12*^ was reduced both in the liver and exocrine pancreas in *fhl1b* MO-injected larvae (D) compared to that of control larvae (C). A-D, confocal projection images, ventral views, anterior to the top. Scale bars, 20 μm.
(TIF)

S12 Fig Forced induction of *fhl1b* during the gastrulation stage directly or indirectly modulates *pdx1* levels for the endocrine and exocrine pancreas development. (A-D) Whole-mount *in situ* hybridization showing the expression of *pdx1* at 18 hpf (A-B) and 48 hpf (C-D), comparing control embryos (A and C) and *fhl1b*-overexpressing embryos (B and D, heat shock applied at 6 hpf). In embryos induced to overexpress *fhl1b* at 6 hpf, both high (white asterisks) and low (gray arrows) levels of *pdx1* expression were reduced at 18 hpf (B). Consistently, at 48 hpf, *pdx1* expression in the principal islet (white dotted circle) and in the developing exocrine pancreas (white bracket) was reduced (D). (E and F) Confocal images of control embryos and embryos induced to overexpress *fhl1b* at 6 hpf, stained for Insulin (red). The number of insulin cells was reduced in *fhl1b*-overexpressing embryos (F), compared to that of control embryos (E) at 78 hpf. (G) The expression of *Tg(ins*:GFP*)*^*zf5*^ (heat shock applied at 6 hpf) was examined at 50 hpf and the percentages of embryos were quantified. The embryos were scored as having a “reduced” expression when the expression of *Tg(ins*:GFP*)*^*zf5*^ was distinctly (> 25%) smaller than that of the control embryos based upon the calculation using ImageJ. A-D, dorsal views, anterior to the top. E-F, confocal projection images, ventral views, anterior to the top. Scale bars, 20 μm.
(TIF)

S13 Fig Fhl1b is an essential mediator of Bmp2b signaling directing the liver versus pancreas fate decision. (A-D) Confocal images of control embryos (A), *bmp2b*-overexpressing embryos (B), *fhl1b* morphants (C), and *bmp2b*-overexpressing *fhl1b* morphants (D) at 72 hpf, stained for Islet (red; expression in the dorsal pancreatic bud is outlined by white dotted circles) and Prox1 (blue). (B) Prox1 expression in the liver was greatly expanded when *bmp2b* expression was induced at the 8-somite stage, whereas Islet expression in the mesenchymal cells surrounding the HPD system as well as in the pancreatic endocrine cells appeared unaffected. As in *fhl1b* morphants (C), the majority of *bmp2b*-overexpressing *fhl1b* morphants exhibited an enlarged Islet-positive pancreatic endocrine cell population with a reduced number of Prox1-positive cells in the liver (D, 80% (22 out of total 28 embryos analyzed)). A small portion of *bmp2b*-overexpressing *fhl1b* morphants restored the developmental defects of the liver and pancreatic endocrine formation (D, 20% (6 out of total 28 embryos analyzed)). (E-L) Whole-mount *in situ* hybridization showing the expression of *hhex* (E-H) and *pdx1* (I-L), comparing control embryos (E and I), *id2a* morphants (F and J), *fhl1b* morphants (G and K), and double *fhl1b*/*id2a* morphants (H and L) at 30 hpf. *hhex* is expressed in the liver (black arrows) and the dorsal pancreatic bud (white dotted circles). *pdx1* is expressed in the developing pancreas including the dorsal pancreatic bud (white dotted circles) and intestine (black brackets), but not in the liver. The *hhex* expression domain was reduced in the liver of *id2a* morphants (F, black arrow) but appeared unaffected in the dorsal pancreatic bud (F, white dotted circle). *fhl1b* morphants showed a reduced *hhex* expression domain in the liver (G, black arrow) with a concomitant expansion of its expression domain in the dorsal pancreatic bud (G, white dotted circle). Double *fhl1b*/*id2a* morphants showed a more severe reduction of *hhex* expression domain in the liver (H, black arrow), whereas its expression domain in the dorsal pancreatic bud was comparable to that in single *fhl1b* morphants (compare G and H, white dotted circles). *pdx1* expression in *id2a* morphants was comparable to that in control embryos (J). *pdx1* expression domain in double *fhl1b*/*id2a* morphants in the dorsal pancreatic bud was expanded (L, white dotted circle), whereas its expression in the intestinal bulb primordium appeared to be reduced (L, black bracket), and was comparable to that in *fhl1b* morphants (K, white dotted circle and black bracket, respectively). (M-N) Whole-mount *in situ* hybridization showing the expression of *id2a*. The expression of *id2a* in the liver biliary epithelial cells of *fhl1b* morphants (N) was comparable to that in control embryos (M) at 72 hpf. (O) Schematic model of the relationship between Bmp2b, Fhl1b, and Id2a in liver versus pancreas fate decision. Fhl1b is essentially required for suppressing *pdx1* expression to keep progenitors competent to differentiate into the liver, while Id2a is required primarily for liver development. Solid lines indicate connections supported by the data previously reported and presented in this study, while dashed lines indicate potential connections by an unknown Bmp2b effector. A-D, confocal projection images, ventral views, anterior to the top. E-N, dorsal views, anterior to the left (n = 20 per each condition). Scale bars, 20 μm.
(TIF)

S14 Fig Fhl1b regulates the capacity of β-cell regeneration. (A) Quantification of the number (mean±SD) of regenerated (Green^+^) and survived β-cells (Yellow^+^; co-expressing green and red) in control, *fhl1b* MO-injected, and *fhl1b*-overexpressing (HS @ 50 hpf) larvae at 36 hours-post-ablation (hpa). Cells in 20 planes of confocal images from 10 individual larvae were counted. (B-C) Confocal images of [*Tg(ins*:*CFP-NTR)*^*s892*^; *Tg(ins*:*Kaede)*^*jh6*^] control (B-B”) and *fhl1b* MO-injected (C-C”) larvae at 24 hpa stained with 2F11 (red) and Carboxypeptidase (blue). A greater number of regenerated β-cells in *fhl1b*-MO injected larvae were mainly located at the junction between the pancreas and the HPD system, specifically at the EPD (C-C”). While upper insets in B’, B”, C’, and C” show the enlarged images of EPD with white arrows pointing the regenerated β-cells, lower insets in B’, B”, C’, and C” only display the magnified images of EPD with white arrows. Abbreviations: GB, gallbladder; CBD, common bile duct; EHD, extrahepatic duct; EPD, extrapancreatic duct; IHD, intrahepatic duct; IPD, intrapancreatic duct. n = 10 per condition. (D) Double antibody and *in situ* hybridization staining of *fhl1b* at 3 dpf in *Tg(ins*:*GFP)*^*zf5*^ embryos. At 3 dpf, the level of *fhl1b* expression is high in the liver (black arrow) and in the distal intestine, low in the HPD system (black bracket), and absent in most pancreatic cells except for a few cells in the principal islet (yellow arrow). In the principal islet, *fhl1b* expression is confined to the peripheral boundary and does not significantly overlap with the core β-cells marked by *Tg(ins*:GFP)^*zf5*^ expression. n = 10. (E-F) Double antibody and *in situ* hybridization staining of *fhl1b* with Somatostatin (E) and Glucagon (F) at 3 dpf in *Tg(ins*:*GFP)*^*zf5*^ embryos. In the principal islet, *fhl1b* expression (black arrowheads in E and F) does not overlap with the Somatostatin-expressing δ-cells (E) but partially with a small number of Glucagon-expressing α-cells (F). *Tg(ins*:GFP*)*^*zf5*^ expression is pseudo colored as white, whereas Somatostatin (E) and Glucagon (F) expression is outlined by both white and black dotted circles. The relative position of *fhl1b*-expressing cells to the Somatostatin (E) and Glucagon (F) expression are indicated by white (E) and yellow (F) arrowheads. Merged views of the middle and right panels are shown in the left panels. n = 10 per condition. (G) Free-glucose levels were measured during β-cell regeneration in wild-type, MTZ-treated, MTZ/*fhl1b* MO-injected, and MTZ/*fhl1b*-overexpressing embryos/larvae. At 7 dpf, free-glucose levels were significantly lower in MTZ/*fhl1b* MO-injected larvae (green line, 512 pmol/larva) than MTZ-treated (red line, 633 pmol/larva) or MTZ/*fhl1b*-overexpressing (purple line, 742 pmol/larva) larvae. *, *P* < 0.05; **, *P* < 0.01; ***, *P* < 0.001. n = 30 larvae (3 pools of 10 larvae) per data point. B, B” and C, C”, confocal projection images. B’ and C’, confocal single-plane images. D-F, confocal single-plane *in situ* hybridization images combined with the projection images of *Tg(ins*:GFP)^*zf5*^ (D), Somatostatin (E), and Glucagon (F) expression. Ventral views, anterior to the top. Scale bars, 20 μm.
(TIF)

S15 Fig Fhl1b blocks induction of late-forming ventral bud-derived endocrine cells. (A-C’) Confocal images of control embryos without SU5402 (A and A’) and with SU5402 (B and B’) treatment as well as *fhl1b*-overexpressing embryos with SU5402 treatment (C and C’, heat shock applied at 50 hpf) at 72 hpf, stained for Islet (red) and Cadherin (blue). Upon treatment of Fgf receptor inhibitor SU5402, ectopic Islet-positive endocrine cells appeared in the hepatopancreatic ductal system (HPD) (B and B’, white asterisks). This effect was blocked by overexpression of *fhl1b* (C and C’). The white lines depict the junction between the pancreas and the HPD. A-C, confocal projection images. A’-C’, confocal single-plane images. Ventral views, anterior to the top. Scale bar, 20 μm.
(TIF)

S1 Table List of genes showing changes in the case of increased Bmp2b signaling. 998 genes with at least 2-fold changes are shown.
(XLSX)

S2 Table List of genes showing changes in the case of decreased Bmp signaling. 1261 genes with at least 2.75-fold changes are shown.
(XLSX)

S3 Table List of genes exhibiting changes in the case of both increased and decreased Bmp signaling. 107 genes with at least 2-fold changes are shown (the genes listed in the S1C Fig are highlighted in blue).
(XLSX)
