# Supplementary figures and images for: Four and a Half LIM Domains 1b (Fhl1b) Is Essential for Regulating the Liver versus Pancreas Fate Decision and for β-Cell Regeneration
Source: PLoS Genet. 2016 Feb 4;12(2):e1005831. doi: 10.1371/journal.pgen.1005831 (PMC4741517; doi:10.1371/journal.pgen.1005831)

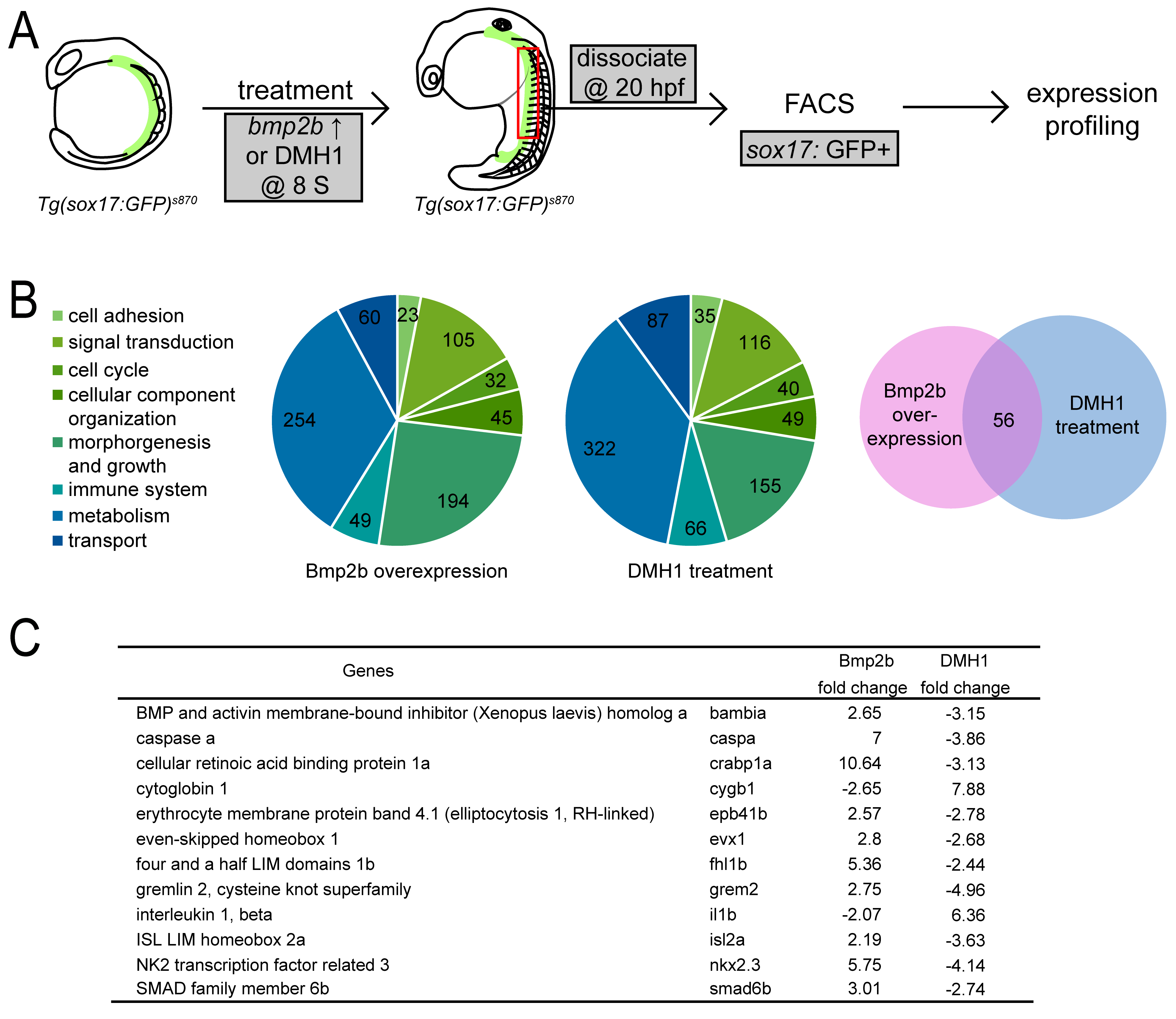

Supplement: S1 Fig — (A) Bmp2b signaling was pharmacologically or genetically manipulated in Tg(sox17:GFP)s870 embryos either by treating with DMH1 or inducing bmp2b expression at the 8-somite stage. Tg(sox17:GFP)s870 -positive endodermal cells from dissected zebrafish trunks containing the organ-forming area (red rectangle) were isolated by FACS and subjected to transcriptome profiling at 20 hpf. (B) Functional clustering and distribution of known genes identified in the expression profiling with a p-value ≤ 0.05 and minimum a 2-fold change in bmp2b overexpressing or a 2.75-fold change in DMH1-treated embryos. Fifty-six known genes showed significant changes in both bmp2b overexpressing and DMH1-treated embryos. (C) List of genes showing prominent changes in both DMH1-treated and bmp2b-overexrpessing conditions. (TIF) [file pgen.1005831.s001.tif]

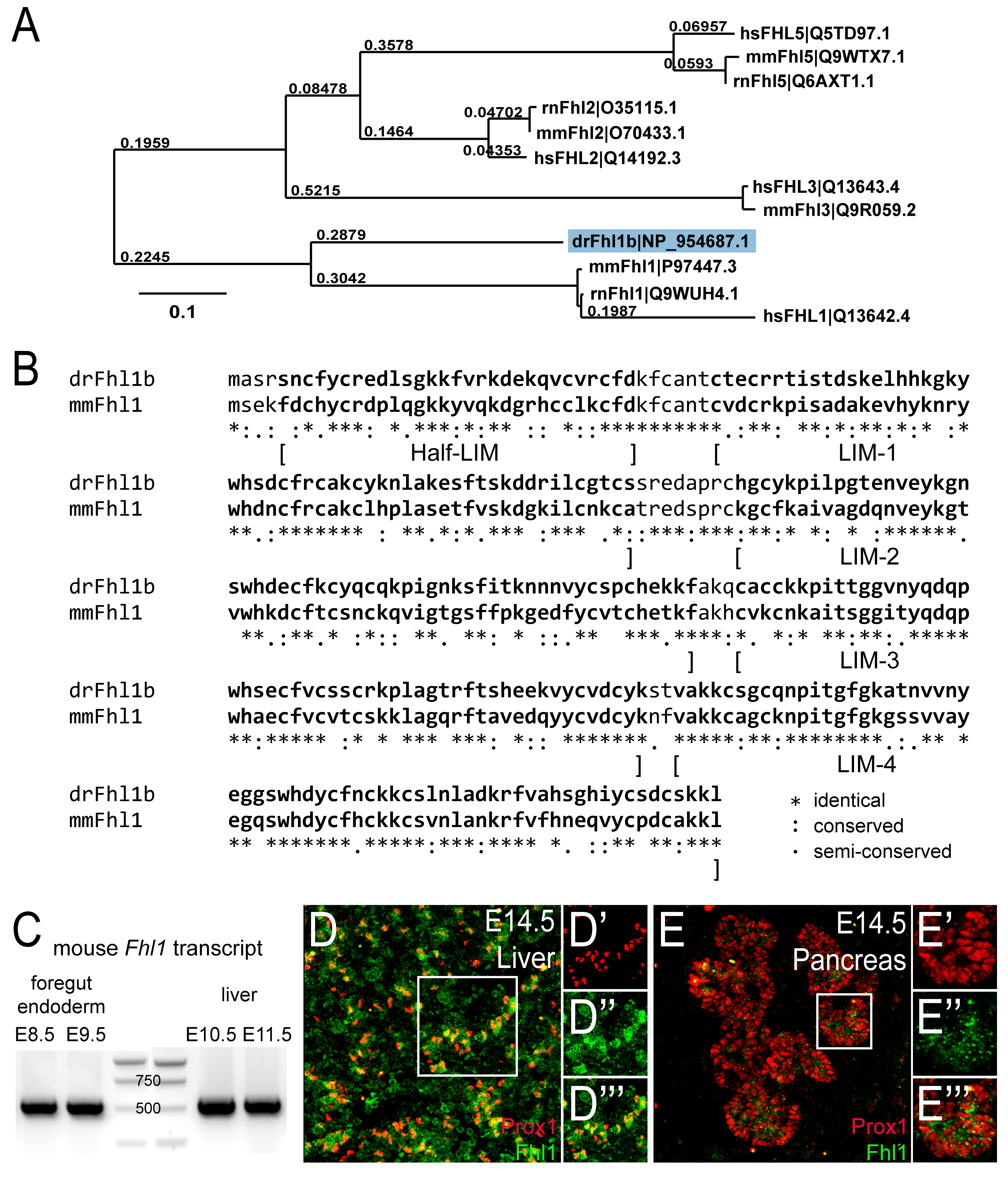

Supplement: S2 Fig — (A) Phylogenetic tree of zebrafish Fhl1b (highlighted in blue) and the related proteins in mammals. This tree was constructed using Phylogeny.fr with sorted candidates from UniProtKB/Swiss-Prot database. Zebrafish (dr), Mouse (mm), Rat (rn), and Human (hs). (B) Alignment of zebrafish Fhl1b and mouse Fhl1 amino acid sequences. Identical residues are indicated with asterisks. (C-E”‘) Expression of Fhl1 in developing mouse embryos. (C) Fhl1 full-length transcript is expressed in the mouse foregut endoderm at embryonic day 8.5 (E8.5)-E9.5. From E10.5, Fhl1 is expressed in the liver. (D-E”‘) Immunofluorescent labeling of Fhl1 in the liver (D-D”‘) and pancreas (E-E”‘) of E14.5 mice (n = 3). (D-D”‘) Fhl1 proteins are highly co-expressed in the Prox1-positive liver cells. (E-E”‘) Fhl1b proteins are weakly detected in the Prox1-positive pancreas cells. To better visualize hepatic and pancreatic Fhl1 expression, magnified images for Prox1 (red; top panel), Fhl1 (green; middle panel), and a merged view (bottom panel) are shown in insets in D’-D”‘ and E’-E”‘, respectively. (TIF) [file pgen.1005831.s002.tif]

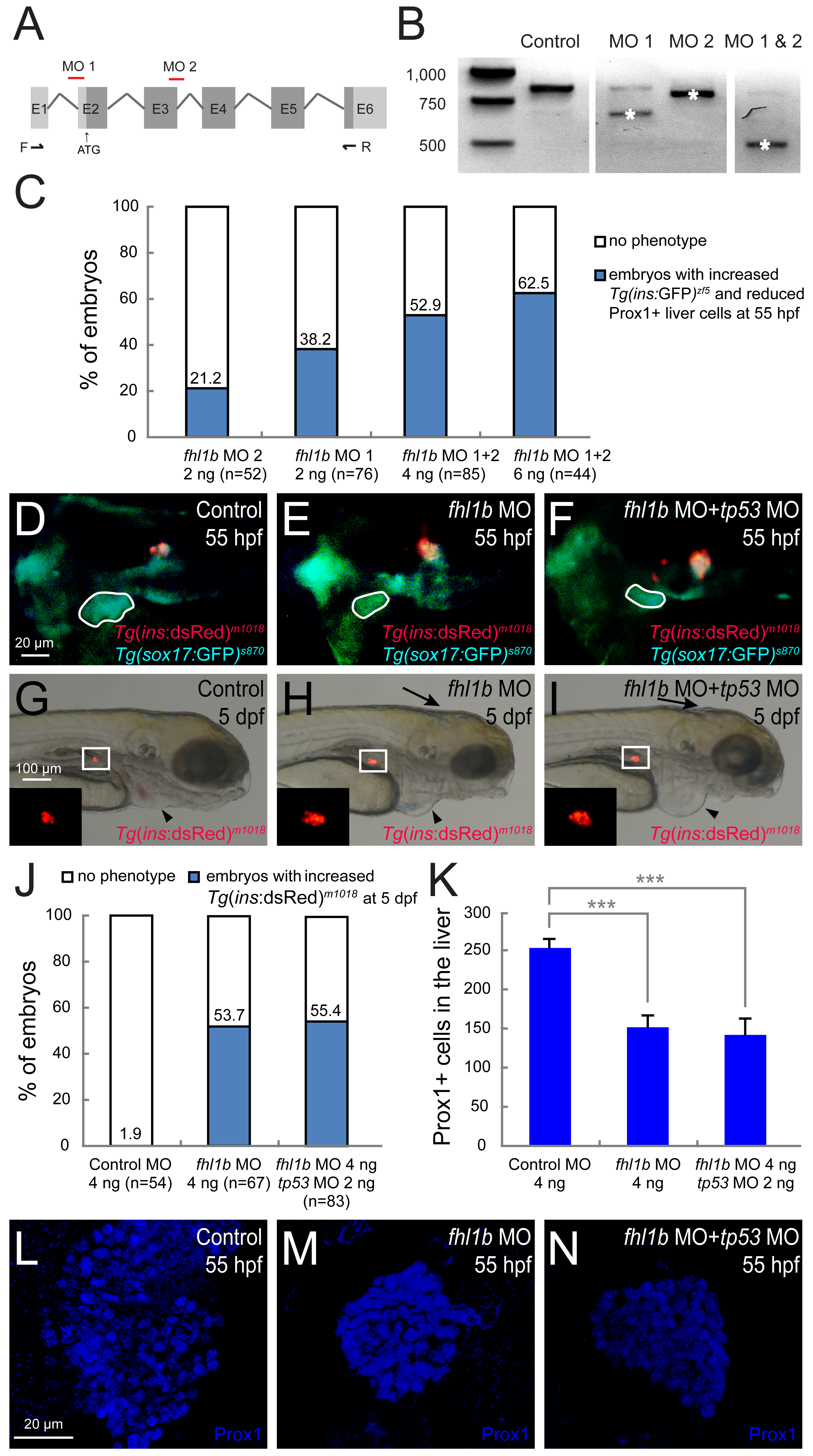

Supplement: S3 Fig — (A) Schematic diagram of fhl1b genomic structure and targeting positions of fhl1b MOs (red lines). Black arrows indicate the position of primers (F and R) used for RT-PCR analysis shown in (B). E1-E6: exon 1 to exon 6. Dark grey, coding regions; Light grey, untranslated regions. (B) RT-PCR analysis of fhl1b knockdown efficiency. Both MO 1 and MO 2 blocked the endogenous splice site of fhl1b and, as a result, either a deletion of exon 2 (MO 1, white asterisk) or a formation of a cryptic splice form of exon 3 (MO 2, white asterisk) occurred, while a combination of MO 1 and 2 led to deletion of both exon 2 and 3 (MO 1 & 2, white asterisk). (C) The percentages of embryos are given for each single MO or combination of MOs based upon the expression domain of Tg(ins:GFP)zf5 in the pancreas and Prox1 in the liver at 55 hpf. The embryos were scored as having a “reduced” or “increased” expression domain when the expression area of each marker was distinctly (> 25%) smaller or larger than that of the control embryos based upon the calculation using ImageJ. (D-F) Fluorescent images of Tg(ins:dsRed)m1018 and Tg(sox17:GFP)s870 expression showing that the developmental defects of the liver (white dotted circles) and β-cell formation in single fhl1b morphants (E) was comparable to double fhl1b/tp53 morphants (F) at 55 hpf (n = 52, control; n = 64, single fhl1b morphants; n = 72, double fhl1b/tp53 morphants). (G-I) Bright-field images combined with fluorescent images showing the overall morphology of embryos and Tg(ins:dsRed)m1018 expression (red) in control (G), single fhl1b morphants (H), and double fhl1b/tp53 morphants (I) at 5 dpf. The enlarged Tg(ins:dsRed)m1018 -expressing cell population (white squares and insets) in single fhl1b morphants (H) was similar to that in embryos co-injected with fhl1b and tp53 MOs (I). Note that potential off-target ventricle lumen inflation defects in the brain of single fhl1b morphants were attenuated by co-knockdown of tp53 (black arrows), wher [file pgen.1005831.s003.tif]

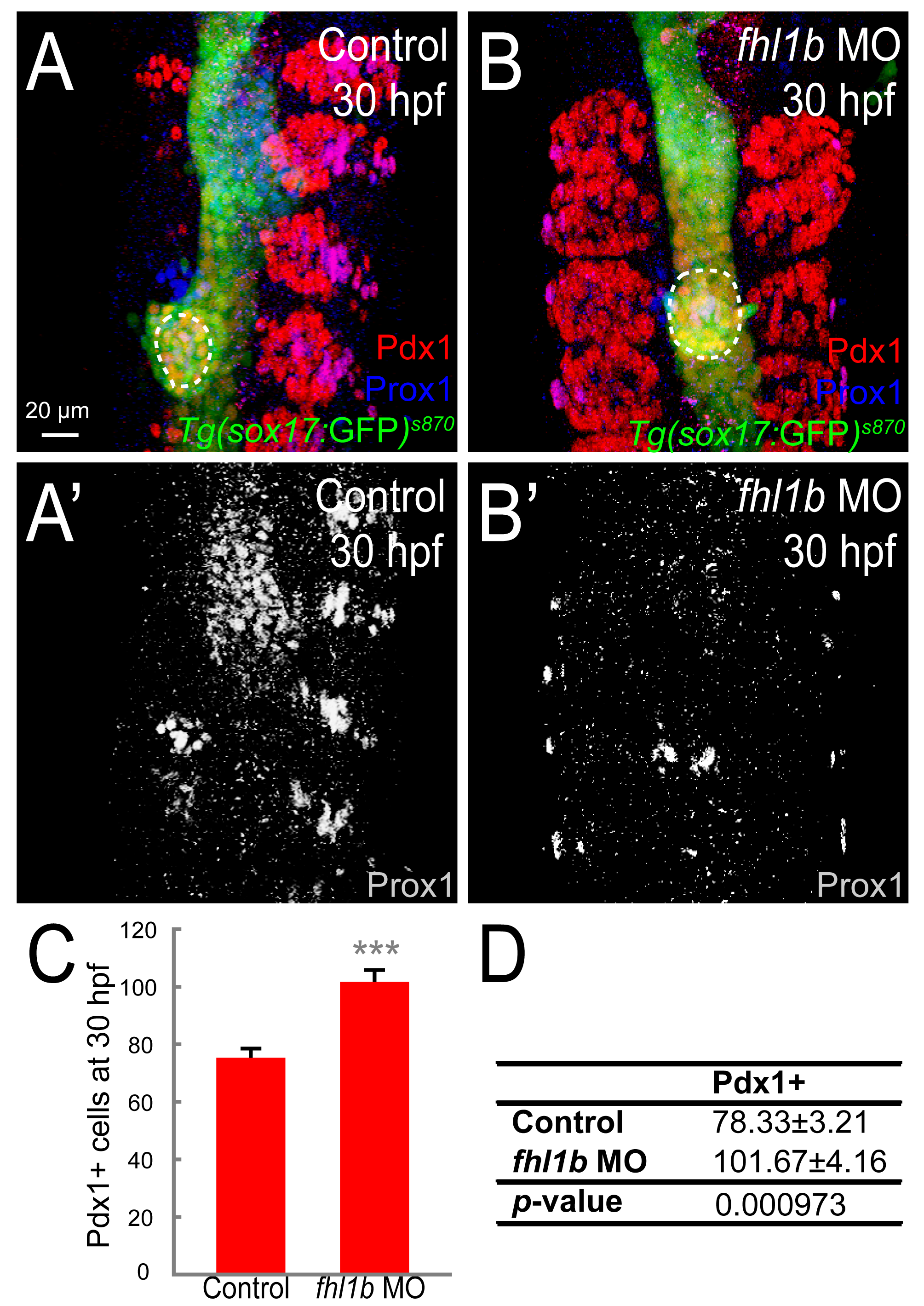

Supplement: S4 Fig — (A-B’) Confocal images of Tg(sox17:GFP)s870 control embryos (A and A′) and fhl1b morphants (B and B′) at 30 hpf, stained for Pdx1 (red; dorsal pancreatic bud is outlined by white dotted circles) and Prox1 (blue in A and B; grey in A’ and B’). The somites are also Pdx1 positive. Compared to control embryos (A and A′), in fhl1b morphants (B and B′), the Pdx1 expression domain in the dorsal pancreatic bud was expanded, while the Prox1 expression domain was significantly reduced. (C-D) Quantification of the number (mean±SD) of Pdx1-positive cells in the pancreas at 30 hpf. Cells in 20 planes of confocal images from 5 individual embryos were counted. Asterisks indicate statistical significance: ***, P < 0.001. A-B’, confocal projection images, ventral views, anterior to the top. Scale bar, 20 μm. (TIF) [file pgen.1005831.s004.tif]

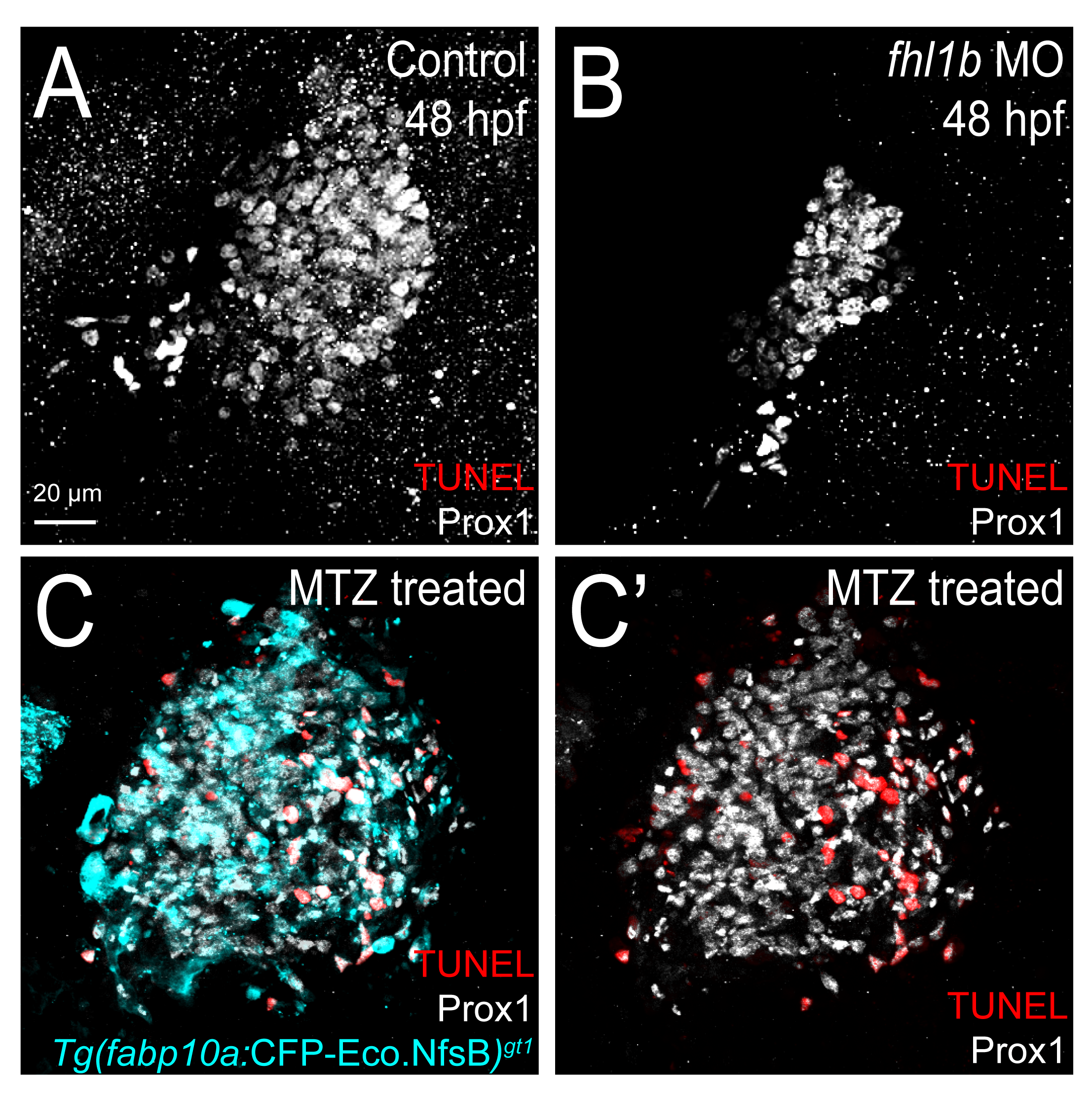

Supplement: S5 Fig — (A-B) TUNEL labeling (red) combined with anti-Prox1 immunostaining (grey) revealed that no TUNEL-positive liver cells were observed both in fhl1b morphants and control embryos at 48 hpf. (C-C’) As a control, Tg(fabp10a:CFP-NTR)gt1 embryos were used. Treating metronidazole (MTZ) caused apoptosis in a large number of hepatocytes. A-C’, confocal projection images, ventral views, anterior to the top. Scale bar, 20 μm. (TIF) [file pgen.1005831.s005.tif]

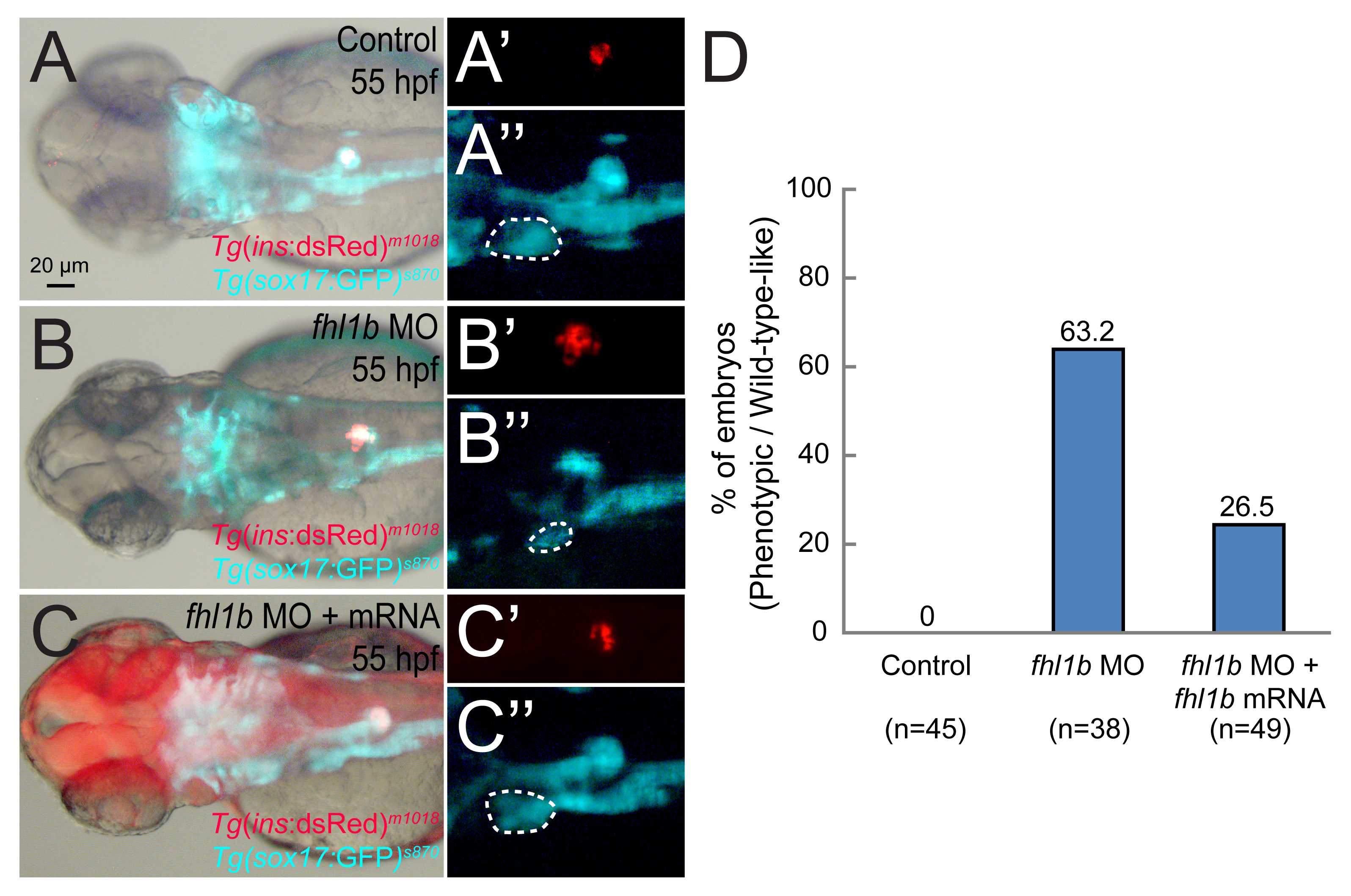

Supplement: S6 Fig — (A-C”) The developmental defects of the liver (A-C and white dotted circles in A”-C”) and β-cell formation (A-C and A’-C’) in fhl1b morphants (B-B”) could be partially rescued by injection of fhl1b-P2A-mcherry mRNA (C-C”), restoring liver size and the β-cell population to a degree comparable to that of control embryos (A-A”) at 55 hpf. Fhl1b translation was monitored by mCherry expression as shown in C. (D) Quantification of the results in A-C”. The embryos were scored as having a “reduced” or “increased” expression domain when the expression area of Tg(ins:dsRed)m1018 and Tg(sox17:GFP)s870 was distinctly (> 25%) smaller or larger than that of the control embryos based upon the calculation using ImageJ. A-C, bright-field images combined with fluorescent image of Tg(ins:dsRed)m1018 and Tg(sox17:GFP)s870 expression. A’-C”, fluorescent images of Tg(ins:dsRed)m1018 and Tg(sox17:GFP)s870 expression. Dorsal views, anterior to the left. Scale bar, 20 μm. (TIF) [file pgen.1005831.s006.tif]

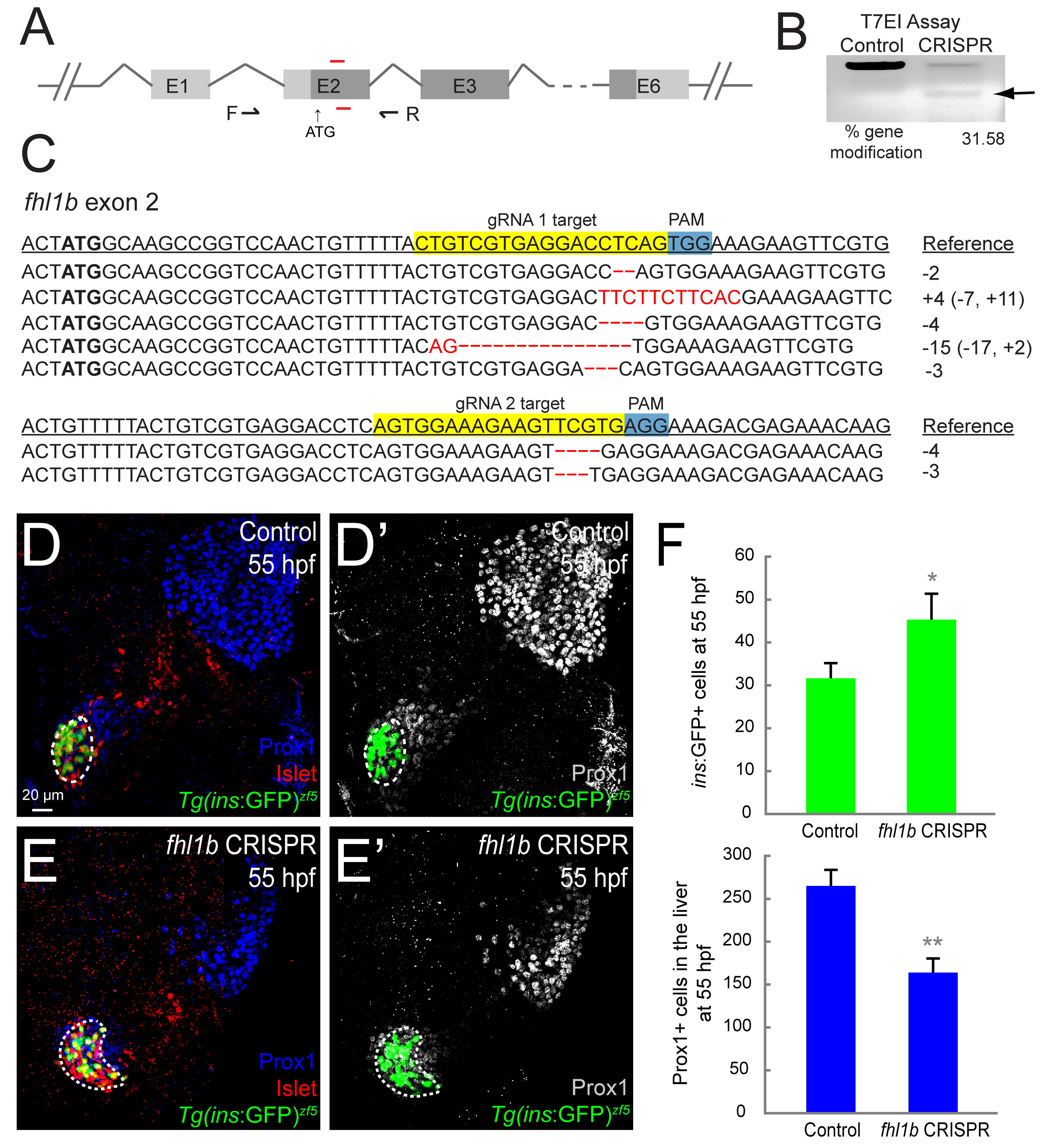

Supplement: S7 Fig — (A) Illustration showing the position of two gRNA-targeting sites (red lines) in the fhl1b locus in zebrafish. Black arrows indicate the position of primers (F and R) used for sequencing to identify indels shown in (C). (B) Representative T7EI assay showing the efficiency of Cas9-mediated cleavage in a single embryo at 55 hpf. (C) Representative Sanger sequencing results of the PCR amplicons of 4 individual embryos at 55 hpf, showing indels induced by Cas9/gRNA in the targeted fhl1b locus. Twenty to thirty clones were sequenced for each embryo. The wild-type sequence is shown at the top with the target sites highlighted in yellow and the PAM sequences (TGG and AGG) highlighted in blue. Deletions are shown as red dashed lines and insertions are highlighted in red. The net change in length caused by each indel is to the right of each sequence (+, insertion; -, deletion). (D-E’) Confocal images of Tg(ins:GFP)zf5 control embryos (D and D’) and Cas9/gRNA-induced mutant embryos (E and E’) at 55 hpf, stained for Prox1 (blue in D and E; grey in D’ and E’) and Islet (red; expression in the dorsal pancreatic bud is outlined by white dotted circles). Cas9/gRNA-induced mutant embryos exhibited an enlarged Insulin-expressing β-cell population with a reduced number of Prox1-positive cells in the liver, phenocopying that of the fhl1b MO knockdown embryos. (F) Quantification of the number (mean±SD) of Insulin-positive cells in the pancreas (green) and Prox1-positive cells in the liver (blue) at 55 hpf. 31.6±3.5 cells were Insulin-positive in control embryos, whereas 45.3±6.0 cells expressed Insulin in Cas9/gRNA-induced mutant embryos. 164±16.5 cells expressed Prox1 in Cas9/gRNA-induced mutant embryos, while 265±18.6 cells were Prox1-positive in control embryos. Cells in 20 planes of confocal images from 5 individual embryos were counted. Asterisks indicate statistical significance: *, P < 0.05, **, P < 0.01. D-E’, confocal projection images, ventral views, anterior to the top. Scal [file pgen.1005831.s007.tif]

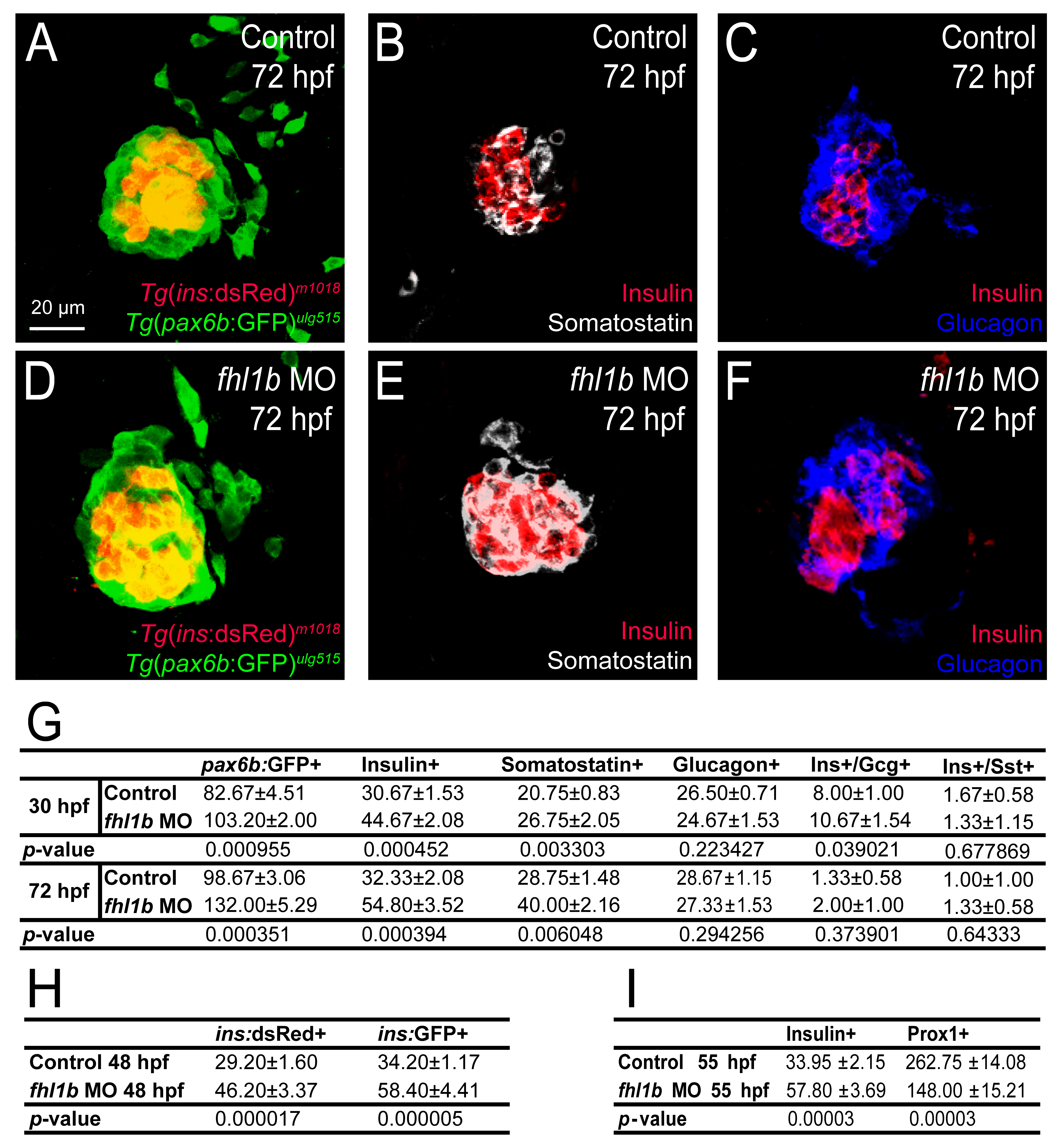

Supplement: S8 Fig — (A-F) Confocal images showing Tg(ins:dsRed)m1018 (A and D) or Insulin (B-C, E-F, red) expression with Tg(P0-pax6b:GFP)ulg515 (A and D, green), Somatostatin (B and E, grey), or Glucagon (C and F, blue) expression at 72 hpf, comparing control embryos (A-C) and fhl1b morphants (D-F). The number of Tg(ins:dsRed)m1018- or Insulin-expressing cells was significantly increased in fhl1b morphants (D-F) compared to that of control embryos (A-C). The number of Tg(P0-pax6b:GFP)ulg515- and Somatostatin-expressing cells was also increased (D and E, respectively), whereas that of Glucagon-expressing cells appeared unaffected (F) in fhl1b morphants compared to control embryos (A, B, and C, respectively). (G) Quantification of the number (mean±SD) of total and individual pancreatic endocrine hormone-expressing cells, comparing control embryos and fhl1b morphants at 30 and 72 hpf. (H) Quantification of the number (mean±SD) of dsRed- and GFP-positive β-cells, comparing control embryos and fhl1b morphants at 48 hpf. (I) Quantification of the number (mean±SD) of Insulin-positive cells in the pancreas and Prox1-positive cells in the liver at 55 hpf. A-F, confocal projection images, ventral views, anterior to the top. G-I, cells in 20 planes of confocal images from 5 individual embryos were counted. Scale bar, 20 μm. (TIF) [file pgen.1005831.s008.tif]

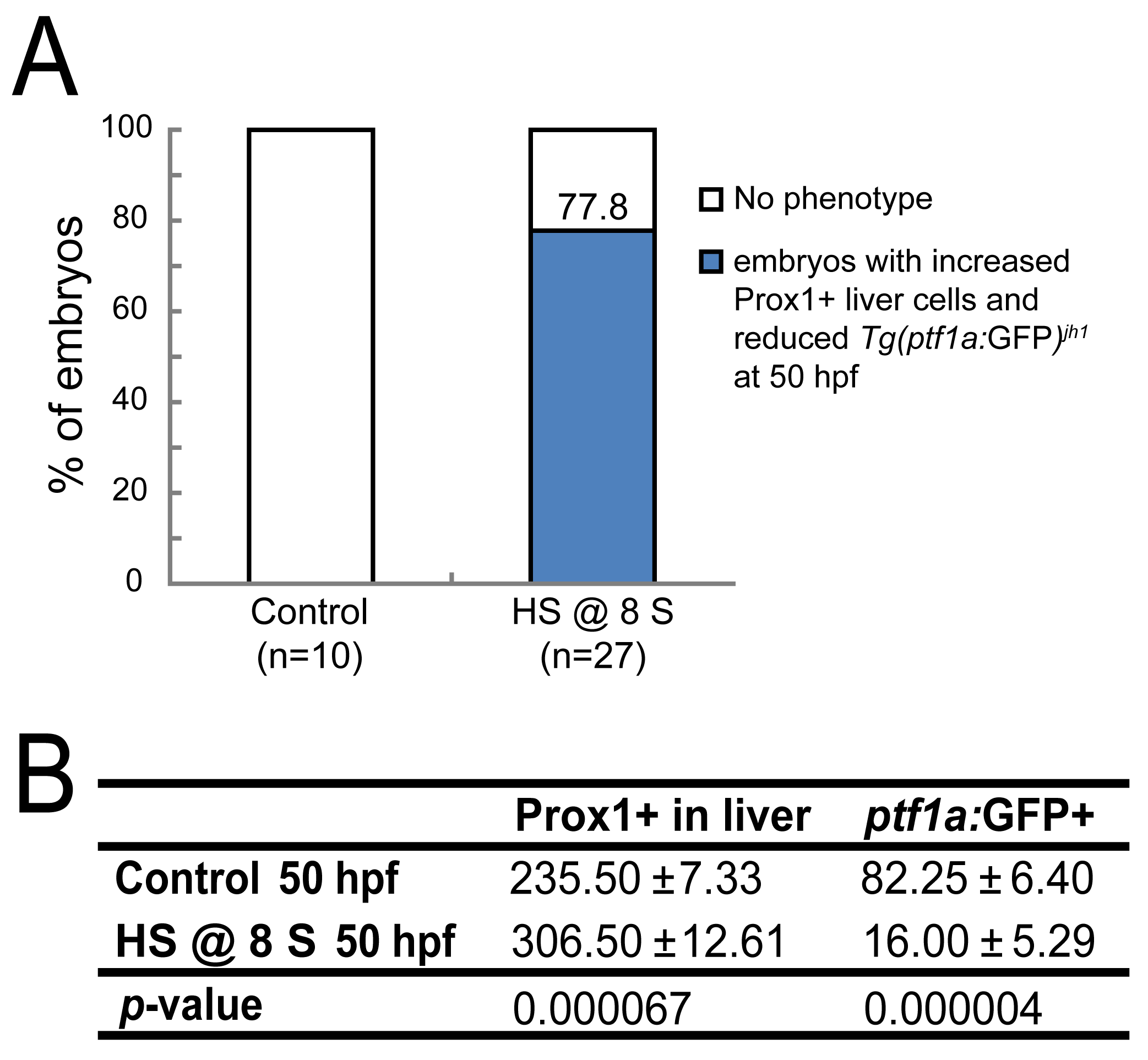

Supplement: S9 Fig — (A) The expression of Tg(ptf1a:GFP)jh1 in the exocrine pancreas and Prox1 in the liver (heat shock applied at the 8-somite stage) was examined at 50 hpf and the percentages of embryos were quantified. The embryos were scored as having a “reduced” or “increased” expression when the expression of each marker was distinctly (> 25%) smaller or larger than that of the control embryos based upon the calculation using ImageJ. (B) Quantification of the number (mean±SD) of Tg(ptf1a:GFP)jh1-positive pancreatic exocrine cells and Prox1-positive cells in the liver, comparing control embryos and fhl1b-overexpressing embryos (heat shock applied at the 8-somite stage) at 50 hpf. Cells in 20 planes of confocal images from 5 individual embryos were counted. (TIF) [file pgen.1005831.s009.tif]

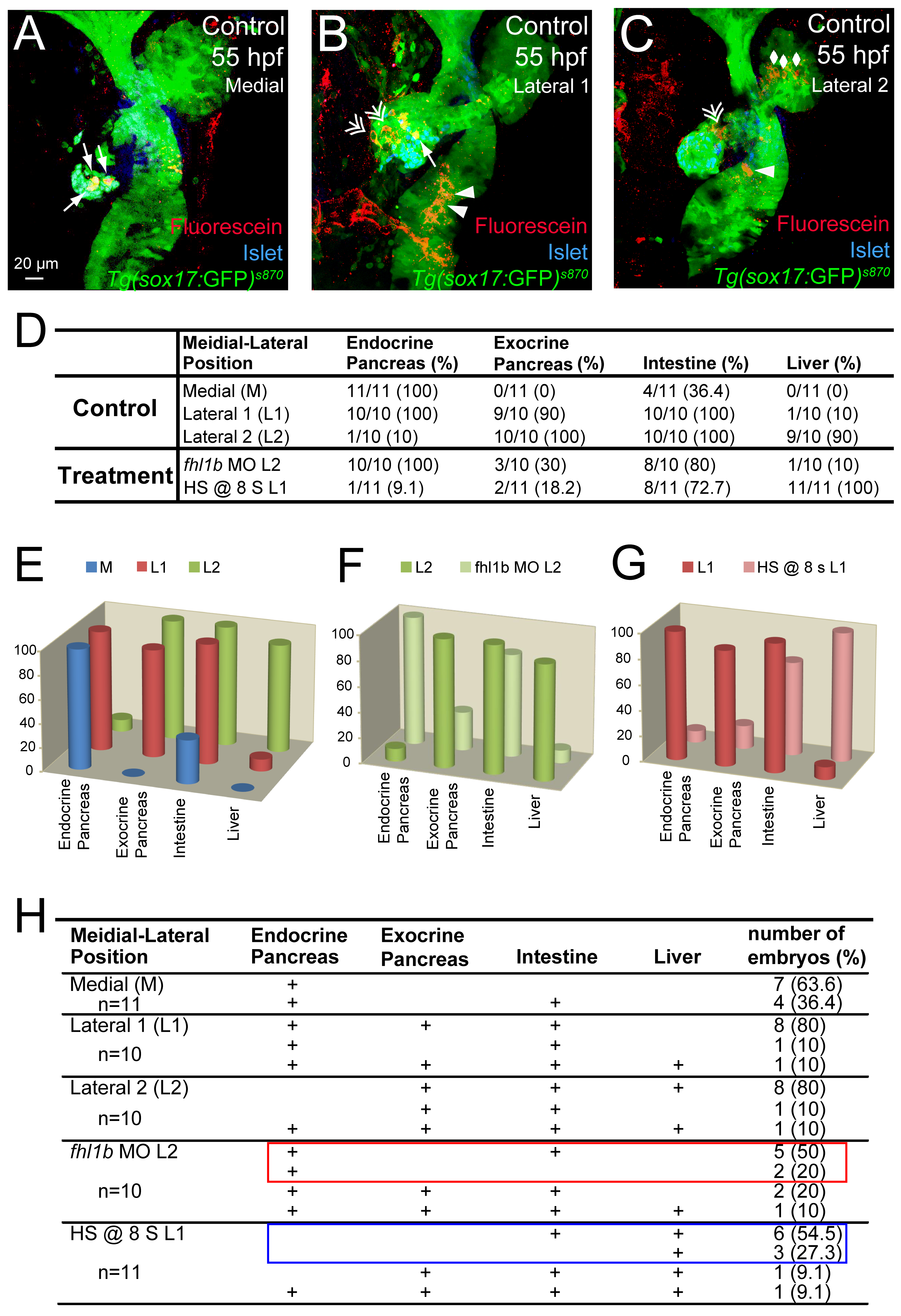

Supplement: S10 Fig — (A-C) Confocal images of Tg(sox17:GFP)s870 embryos at 55 hpf, stained for Islet (blue) and uncaged-Fluorescein (red), showing the progeny of the medial (A), lateral 1 (B) and lateral 2 (C) cells. Medial cells (A) mostly gave rise to pancreatic endocrine cells (white arrows). Lateral 1 cells (B) gave rise to pancreatic exocrine (white double arrows), endocrine (white arrow), and intestinal (white arrowheads) cells. Lateral 2 cells (C) gave rise to liver (white rhombi), intestine (white arrowhead), and pancreatic exocrine cells (white double arrow). (D-H) The numbers and the percentages of embryos that showed incorporation into a given tissue type in each specific position, comparing control embryos (D, E, F (as L2), G (as L1), and H) as well as fhl1b morphants and embryos induced to overexpress fhl1b at the 8-somite stage (D, F (as fhl1b MO L2), G (as HS @ 8s L1), and H). In every fhl1b-depleted embryo, lateral 2 cells contributed to the pancreatic endocrine cells (D, F (as fhl1b MO L2), and H), while in control embryos, most of the lateral 2 cells gave rise to the exocrine pancreas, intestine, and liver, but seldom gave rise to the endocrine pancreas (D, E, F (as L2), and H). In control embryos, lateral 1 cells mostly gave rise to pancreatic and intestinal cells, but not to liver cells (D, E, G (as L1), and H), whereas in every fhl1b-overexpressing embryo, lateral 1 cells contributed to the liver (D, G (as HS @ 8s L1), and H). Data in each 3-D column (%) in E-G were obtained by summing the number of embryos that showed incorporation into a given tissue type and normalizing it to the total number of embryos examined in each specific position: M, L1 and L2. Colored rectangles in H highlight the most dominant pattern in fhl1b morphants (red) and fhl1b-overexpressing (blue) embryos. A-C, confocal projection images, ventral views, anterior to the top. Scale bar, 20 μm. (TIF) [file pgen.1005831.s010.tif]

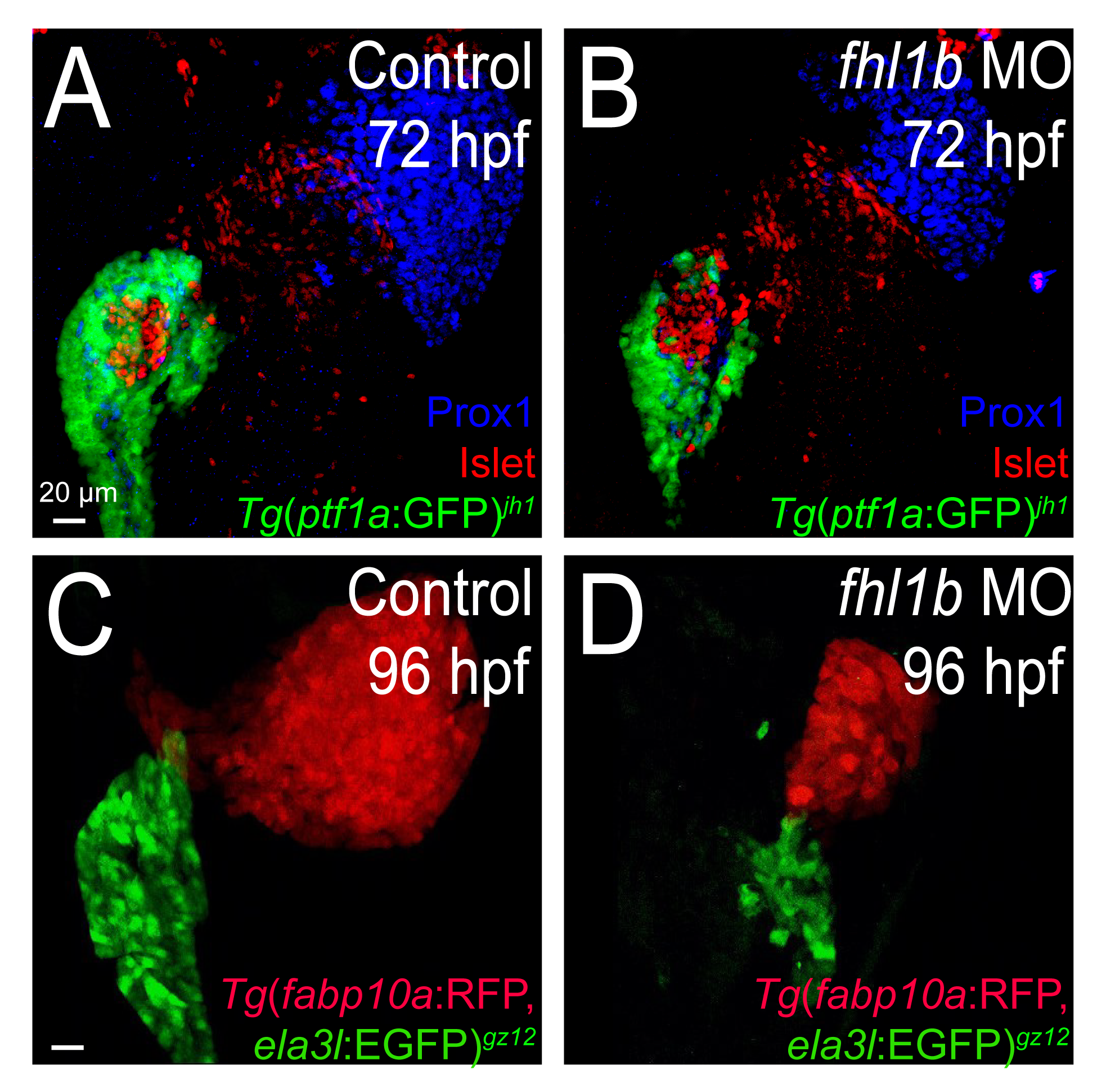

Supplement: S11 Fig — (A and B) Confocal images of Tg(ptf1a:GFP)jh1 control embryos (A) and fhl1b morphants (B), stained for Prox1 (blue) and Islet (red). The expression domain of the Prox1 and Tg(ptf1a:GFP)jh1 was reduced in fhl1b morphants (B) compared to that of control embryos (A). (C and D) Confocal images of Tg(fabp10a:RFP, ela3l:EGFP)gz12 control embryos (C) and fhl1b MO-injected larvae (D) at 96 hpf. The expression domain of the Tg(fabp10a:RFP, ela3l:EGFP)gz12 was reduced both in the liver and exocrine pancreas in fhl1b MO-injected larvae (D) compared to that of control larvae (C). A-D, confocal projection images, ventral views, anterior to the top. Scale bars, 20 μm. (TIF) [file pgen.1005831.s011.tif]

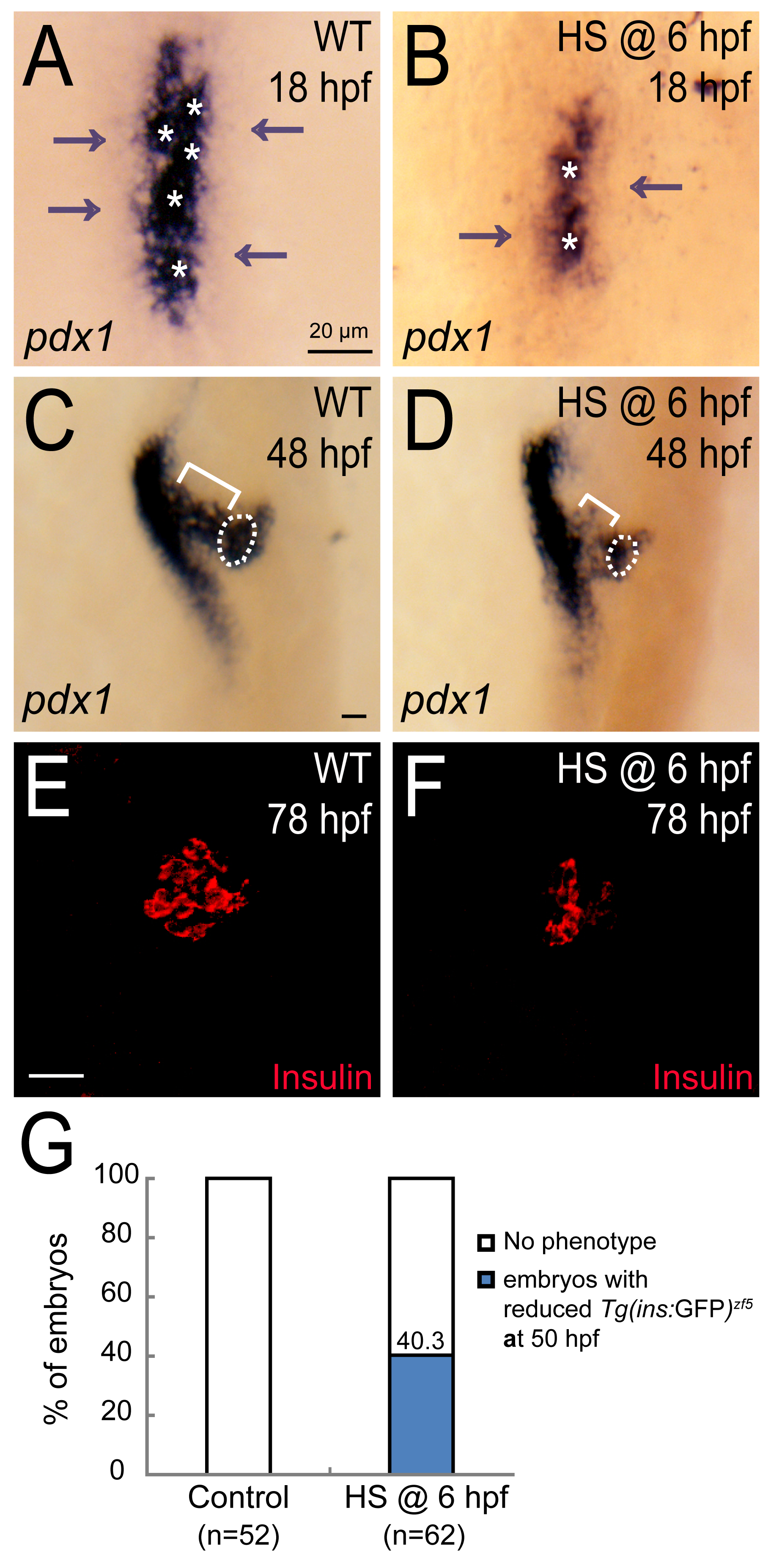

Supplement: S12 Fig — (A-D) Whole-mount in situ hybridization showing the expression of pdx1 at 18 hpf (A-B) and 48 hpf (C-D), comparing control embryos (A and C) and fhl1b-overexpressing embryos (B and D, heat shock applied at 6 hpf). In embryos induced to overexpress fhl1b at 6 hpf, both high (white asterisks) and low (gray arrows) levels of pdx1 expression were reduced at 18 hpf (B). Consistently, at 48 hpf, pdx1 expression in the principal islet (white dotted circle) and in the developing exocrine pancreas (white bracket) was reduced (D). (E and F) Confocal images of control embryos and embryos induced to overexpress fhl1b at 6 hpf, stained for Insulin (red). The number of insulin cells was reduced in fhl1b-overexpressing embryos (F), compared to that of control embryos (E) at 78 hpf. (G) The expression of Tg(ins:GFP)zf5 (heat shock applied at 6 hpf) was examined at 50 hpf and the percentages of embryos were quantified. The embryos were scored as having a “reduced” expression when the expression of Tg(ins:GFP)zf5 was distinctly (> 25%) smaller than that of the control embryos based upon the calculation using ImageJ. A-D, dorsal views, anterior to the top. E-F, confocal projection images, ventral views, anterior to the top. Scale bars, 20 μm. (TIF) [file pgen.1005831.s012.tif]

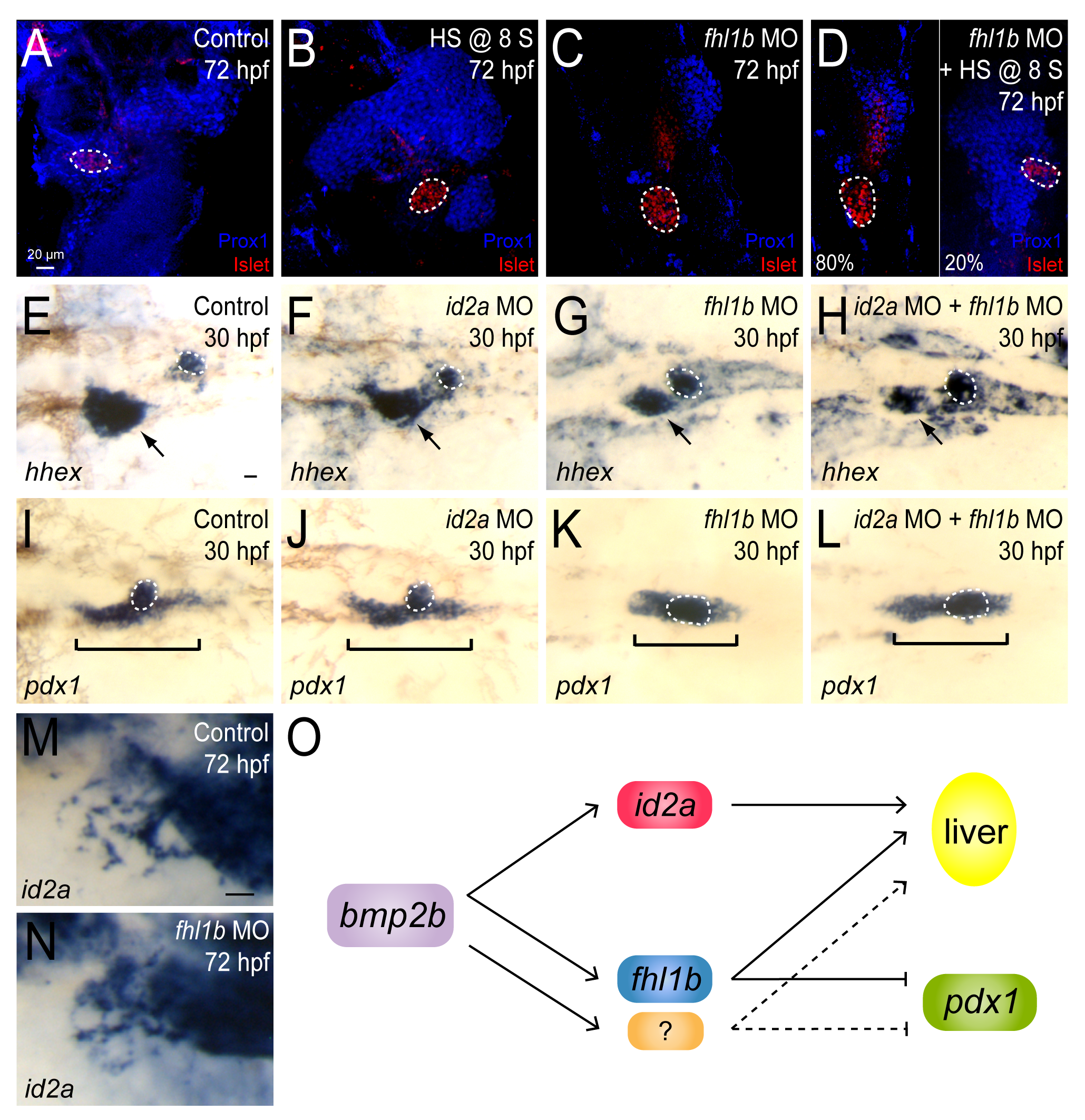

Supplement: S13 Fig — (A-D) Confocal images of control embryos (A), bmp2b-overexpressing embryos (B), fhl1b morphants (C), and bmp2b-overexpressing fhl1b morphants (D) at 72 hpf, stained for Islet (red; expression in the dorsal pancreatic bud is outlined by white dotted circles) and Prox1 (blue). (B) Prox1 expression in the liver was greatly expanded when bmp2b expression was induced at the 8-somite stage, whereas Islet expression in the mesenchymal cells surrounding the HPD system as well as in the pancreatic endocrine cells appeared unaffected. As in fhl1b morphants (C), the majority of bmp2b-overexpressing fhl1b morphants exhibited an enlarged Islet-positive pancreatic endocrine cell population with a reduced number of Prox1-positive cells in the liver (D, 80% (22 out of total 28 embryos analyzed)). A small portion of bmp2b-overexpressing fhl1b morphants restored the developmental defects of the liver and pancreatic endocrine formation (D, 20% (6 out of total 28 embryos analyzed)). (E-L) Whole-mount in situ hybridization showing the expression of hhex (E-H) and pdx1 (I-L), comparing control embryos (E and I), id2a morphants (F and J), fhl1b morphants (G and K), and double fhl1b/id2a morphants (H and L) at 30 hpf. hhex is expressed in the liver (black arrows) and the dorsal pancreatic bud (white dotted circles). pdx1 is expressed in the developing pancreas including the dorsal pancreatic bud (white dotted circles) and intestine (black brackets), but not in the liver. The hhex expression domain was reduced in the liver of id2a morphants (F, black arrow) but appeared unaffected in the dorsal pancreatic bud (F, white dotted circle). fhl1b morphants showed a reduced hhex expression domain in the liver (G, black arrow) with a concomitant expansion of its expression domain in the dorsal pancreatic bud (G, white dotted circle). Double fhl1b/id2a morphants showed a more severe reduction of hhex expression domain in the liver (H, black arrow), whereas its expression domain in the dorsal pancrea [file pgen.1005831.s013.tif]

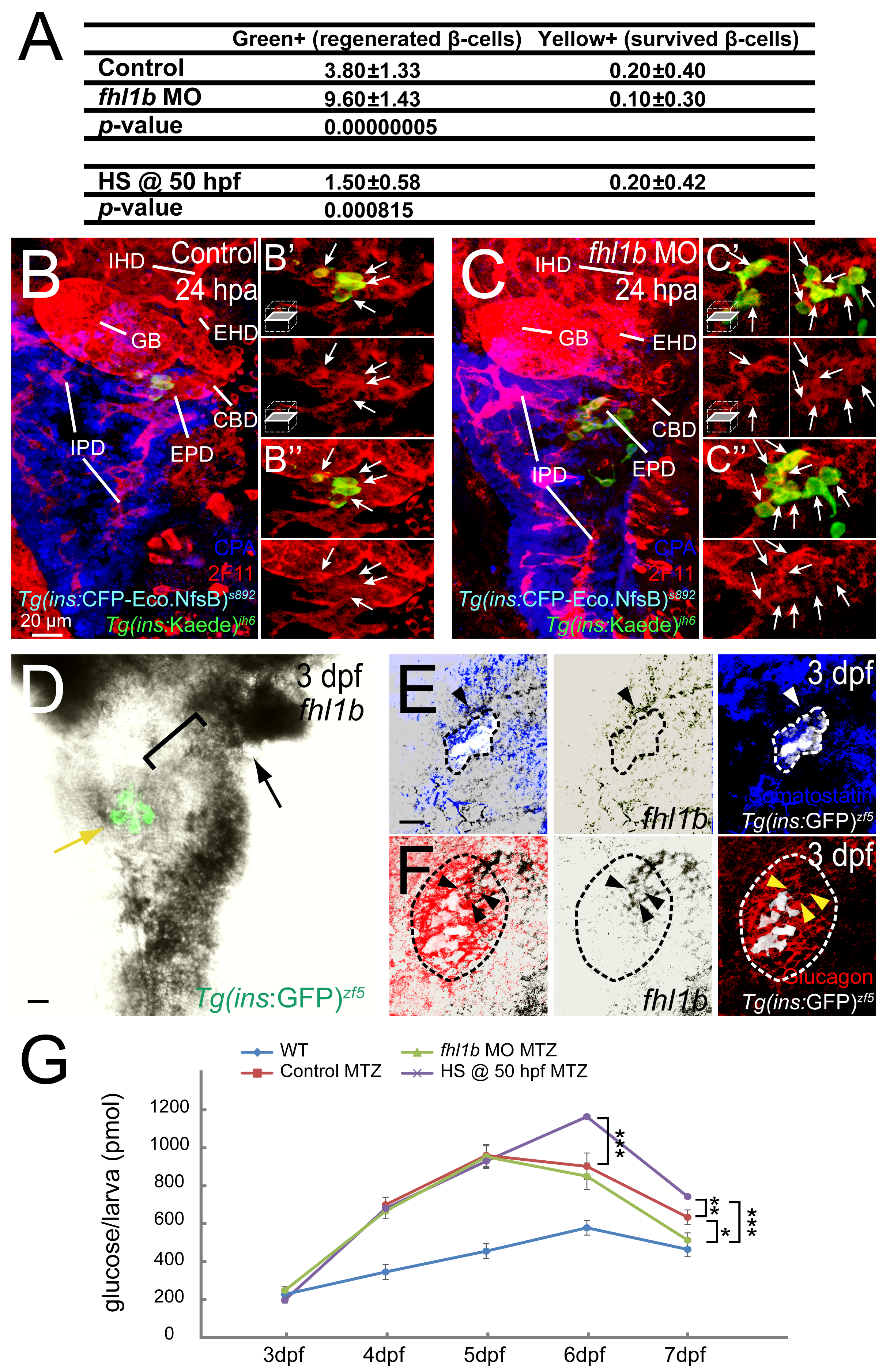

Supplement: S14 Fig — (A) Quantification of the number (mean±SD) of regenerated (Green+) and survived β-cells (Yellow+; co-expressing green and red) in control, fhl1b MO-injected, and fhl1b-overexpressing (HS @ 50 hpf) larvae at 36 hours-post-ablation (hpa). Cells in 20 planes of confocal images from 10 individual larvae were counted. (B-C) Confocal images of [Tg(ins:CFP-NTR)s892; Tg(ins:Kaede)jh6] control (B-B”) and fhl1b MO-injected (C-C”) larvae at 24 hpa stained with 2F11 (red) and Carboxypeptidase (blue). A greater number of regenerated β-cells in fhl1b-MO injected larvae were mainly located at the junction between the pancreas and the HPD system, specifically at the EPD (C-C”). While upper insets in B’, B”, C’, and C” show the enlarged images of EPD with white arrows pointing the regenerated β-cells, lower insets in B’, B”, C’, and C” only display the magnified images of EPD with white arrows. Abbreviations: GB, gallbladder; CBD, common bile duct; EHD, extrahepatic duct; EPD, extrapancreatic duct; IHD, intrahepatic duct; IPD, intrapancreatic duct. n = 10 per condition. (D) Double antibody and in situ hybridization staining of fhl1b at 3 dpf in Tg(ins:GFP)zf5 embryos. At 3 dpf, the level of fhl1b expression is high in the liver (black arrow) and in the distal intestine, low in the HPD system (black bracket), and absent in most pancreatic cells except for a few cells in the principal islet (yellow arrow). In the principal islet, fhl1b expression is confined to the peripheral boundary and does not significantly overlap with the core β-cells marked by Tg(ins:GFP)zf5 expression. n = 10. (E-F) Double antibody and in situ hybridization staining of fhl1b with Somatostatin (E) and Glucagon (F) at 3 dpf in Tg(ins:GFP)zf5 embryos. In the principal islet, fhl1b expression (black arrowheads in E and F) does not overlap with the Somatostatin-expressing δ-cells (E) but partially with a small number of Glucagon-expressing α-cells (F). Tg(ins:GFP)zf5 expression is pseudo colored as white, whereas S [file pgen.1005831.s014.tif]

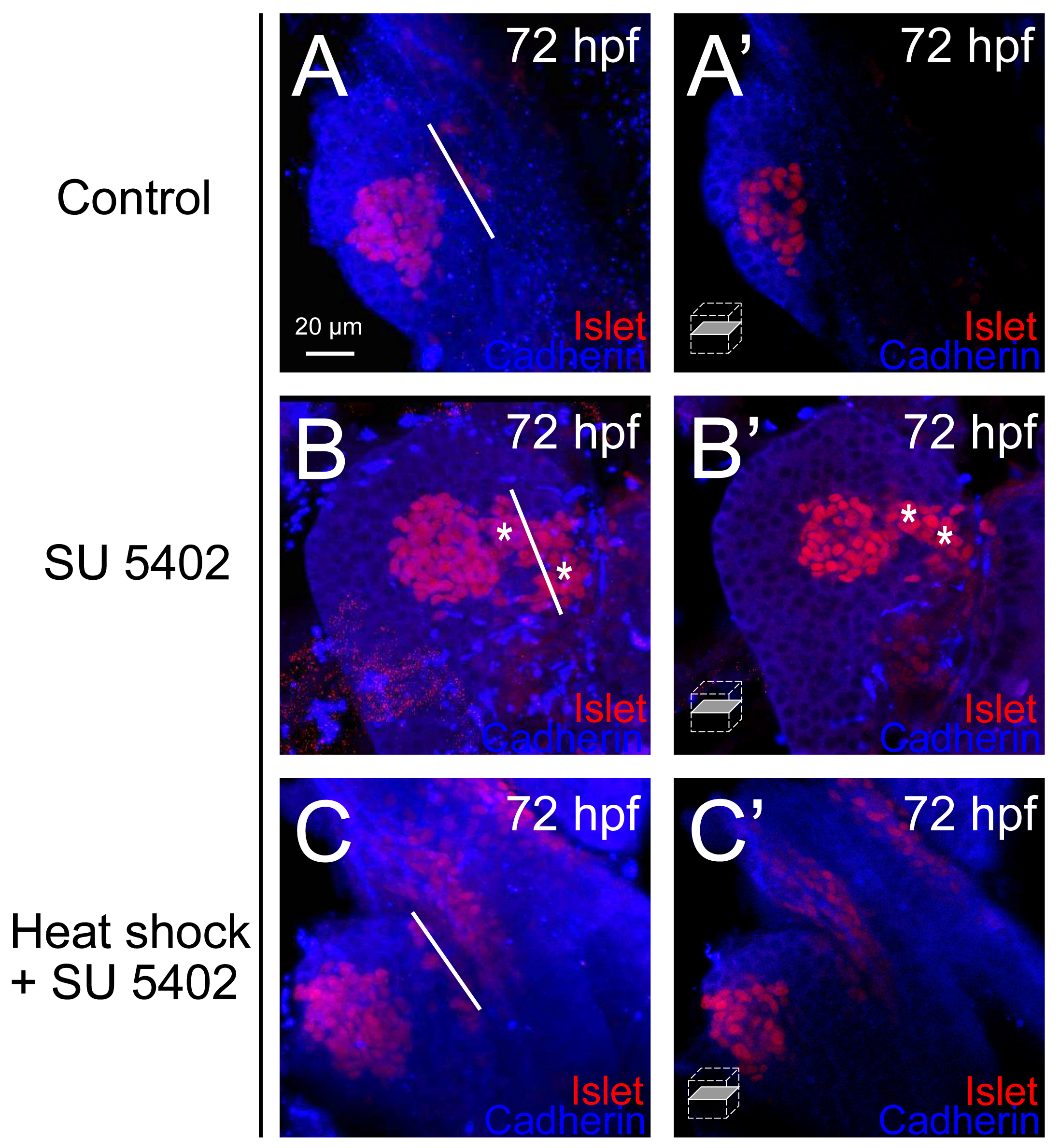

Supplement: S15 Fig — (A-C’) Confocal images of control embryos without SU5402 (A and A’) and with SU5402 (B and B’) treatment as well as fhl1b-overexpressing embryos with SU5402 treatment (C and C’, heat shock applied at 50 hpf) at 72 hpf, stained for Islet (red) and Cadherin (blue). Upon treatment of Fgf receptor inhibitor SU5402, ectopic Islet-positive endocrine cells appeared in the hepatopancreatic ductal system (HPD) (B and B’, white asterisks). This effect was blocked by overexpression of fhl1b (C and C’). The white lines depict the junction between the pancreas and the HPD. A-C, confocal projection images. A’-C’, confocal single-plane images. Ventral views, anterior to the top. Scale bar, 20 μm. (TIF) [file pgen.1005831.s015.tif]
